# *Ambra1* haploinsufficiency in CD1 mice results in metabolic alterations and exacerbates age-associated retinal degeneration

**DOI:** 10.1080/15548627.2022.2103307

**Published:** 2022-07-24

**Authors:** Ignacio Ramírez-Pardo, Beatriz Villarejo-Zori, Juan Ignacio Jiménez-Loygorri, Elena Sierra-Filardi, Sandra Alonso-Gil, Guillermo Mariño, Pedro de la Villa, Patrick S Fitze, José Manuel Fuentes, Ramón García-Escudero, Deborah A. Ferrington, Raquel Gomez-Sintes, Patricia Boya

**Affiliations:** a Department of Cellular and Molecular Biology, Centro de Investigaciones Biológicas Margarita Salas, CSIC, Madrid, Spain; b Department of Functional Biology, University of Oviedo, Spain; c Department of Systems Biology, University of Alcalá, Alcalá de Henares, Madrid, Spain; d Vision neurophisiology group, Instituto Ramón y Cajal de Investigación Sanitaria (IRYCIS), Madrid, Spain; e Departament of Biodiversity and Evolutionary Biology, Museo Nacional de Ciencias Naturales, CSIC, Madrid, Spain; f Department of Biochemistry, Molecular Biology and Genetics, Faculty of Nursing and Occupational Therapy, University of Extremadura, Cáceres, Spain; g Centro de Investigación Biomédica en Red de Enfermedades Neurodegenerativas (CIBERNED), Madrid, Spain; h Nerodegenerative Diseases unit, Instituto Universitario de Investigación Biosanitaria de Extremadura (INUBE), Cáceres, Spain; i Molecular Oncology Unit, CIEMAT, Madrid, Spain; j Biomedical Research Institute I+12, University Hospital 12 de Octubre, Madrid, Spain; k Centro de Investigación Biomédica en Red de Cáncer (CIBERONC), Madrid, Spain; l Department of Ophthalmology and Visual Neurosciences, University of Minnesota, Minneapolis, MN, USA

**Keywords:** Aging, AMBRA1, autophagy, metabolic alterations, neurodegeneration, retina, retinal pigment epithelium

## Abstract

Macroautophagy/autophagy is a key process in the maintenance of cellular homeostasis. The age-dependent decline in retinal autophagy has been associated with photoreceptor degeneration. Retinal dysfunction can also result from damage to the retinal pigment epithelium (RPE), as the RPE-retina constitutes an important metabolic ecosystem that must be finely tuned to preserve visual function. While studies of mice lacking essential autophagy genes have revealed a predisposition to retinal degeneration, the consequences of a moderate reduction in autophagy, similar to that which occurs during physiological aging, remain unclear. Here, we described a retinal phenotype consistent with accelerated aging in mice carrying a haploinsufficiency for *Ambra1*, a pro-autophagic gene. These mice showed protein aggregation in the retina and RPE, metabolic underperformance, and premature vision loss. Moreover, *Ambra1^+/gt^* mice were more prone to retinal degeneration after RPE stress. These findings indicate that autophagy provides crucial support to RPE-retinal metabolism and protects the retina against stress and physiological aging.

**Abbreviations :** 4-HNE: 4-hydroxynonenal; AMBRA1: autophagy and beclin 1 regulator 1, AMD: age-related macular degeneration;; GCL: ganglion cell layer; GFAP: glial fibrillary acidic protein; GLUL: glutamine synthetase/glutamate-ammonia ligase; HCL: hierarchical clustering; INL: inner nuclear layer; IPL: inner plexiform layer; LC/GC-MS: liquid chromatography/gas chromatography–mass spectrometry; MA: middle-aged; MTDR: MitoTracker Deep Red; MFI: mean fluorescence intensity; NL: NH_4_Cl and leupeptin; Nqo: NAD(P)H quinone dehydrogenase; ONL: outer nuclear layer; OPL: outer plexiform layer; OP: oscillatory potentials; OXPHOS: oxidative phosphorylation; PCR: polymerase chain reaction; PRKC/PKCα: protein kinase C; POS: photoreceptor outer segment; RGC: retinal ganglion cells; RPE: retinal pigment epithelium; SI: sodium iodate; TCA: tricarboxylic acid.

## Introduction

The eye is a very metabolically active tissue with high levels of nutrient and oxygen consumption. The avascular outer retina relies on nutrient diffusion from the choroid vessels through the retinal pigment epithelium (RPE), which supplies the retina with nutrients such as glucose [1,2]. This glucose is then metabolized via the tricarboxylic acid (TCA) cycle, oxidative phosphorylation (OXPHOS), and aerobic glycolysis to produce glycerol and ATP, which in turn support phototransduction and phospholipid synthesis to renew photoreceptor outer segments (POS) [3]. β-oxidation of fatty acids and lactate are the main energy sources of the RPE, and are derived from the digestion of POS and photoreceptor glycolysis, respectively [4]. This arrangement results in a high degree of metabolic interdependence between the neural retina and RPE, whereby glucose is converted into lactate in the retina and then shunted to the RPE [5]. The metabolic ecosystem resulting from this interdependence between the two tissue types is key to the preservation of vision [5].

Autophagy is crucial to preserve cell homeostasis, serves as a recycling mechanism to extract nutrients and eliminate damaged cell components, and has important implications in neurological diseases [6,7]. While studies are beginning to unravel the role of autophagy in the eye and the consequences of autophagy alterations in ocular and retinal diseases, the degree of retinal degeneration depends on several factors including the protein that is altered, the cell type affected, and the age of the animal [8,9]. We previously demonstrated a progressive, age-dependent decrease in autophagic flux in the retina of C57/BL6 mice, beginning at 12 months of age [10]. In addition, complete autophagy deficiency, achieved by deleting *Atg5* in neuronal precursors, results in severe retinal degeneration and vision loss at 7 weeks of age [10] while *Atg5* deletion in only cones or rods results in much more subtle phenotypes [8,11,12]. Two mouse models that completely lack the essential autophagy proteins ATG5 or ATG7 in the RPE show increased predisposition to age-related retinal degeneration [13]. However, other studies in which these same autophagy regulators were eliminated in the mouse RPE reported no retinal degeneration [14,15], indicating that further research is needed to determine whether complete autophagy blockade in the RPE results in reduced visual function. More importantly, it is unclear how slight reductions in autophagy in both the RPE and retina, as occurs during physiological aging, affect retinal metabolism and visual function.

AMBRA1 (autophagy and beclin 1 regulator 1) is a multifunctional scaffold protein that promotes autophagy initiation by triggering interaction between BECN1 and PIK3C3/VPS34 [16,17] and ULK1 activity in a MTOR-dependent manner [18]. *Ambra1*-deficient (*Ambra1^gt/gt^*) mice display embryonic lethality and show massive alterations in the central nervous system (CNS) including exencephaly and spina bifida [16], as well as neurogenesis alterations in the olfactory bulb and sub-ventricular zone [19,20]. Moreover, decreased *Ambra1* expression is linked to an attenuated autophagic response that may be implicated in neural disorders and neurodegenerative diseases such as autism, schizophrenia, and Alzheimer disease [21–23]. Recent findings indicate that AMBRA1 mediates an autophagy-mediated protective response to injury in retinal ganglion cells (RGC) [24,25]. It remains to be determined how *Ambra1* deficiency affects the retina-RPE ecosystem in the context of aging.

Here, we showed that monoallelic deletion of *Ambra1* resulted in diminished autophagic flux and induced protein aggregation, lipid peroxidation, and oxidative stress in the retina and RPE. Loss of autophagy-dependent proteostasis led to exacerbated RPE degeneration at 1 year of age and retinal neurodegeneration in 2-year-old mice, culminating in premature vision loss. Moreover, autophagy reduction was associated with metabolic imbalance in *Ambra1^+/gt^* mice, including mitochondrial defects, altered glycolysis, and energetic underperformance. These data demonstrate for the first time that partial loss of autophagy results in marked metabolic alterations and increased susceptibility to RPE stress and age-dependent retinal degeneration.

## Results

### Age-dependent vision loss is exacerbated in *Ambra1^+/gt^* mice

**Figure 1.**
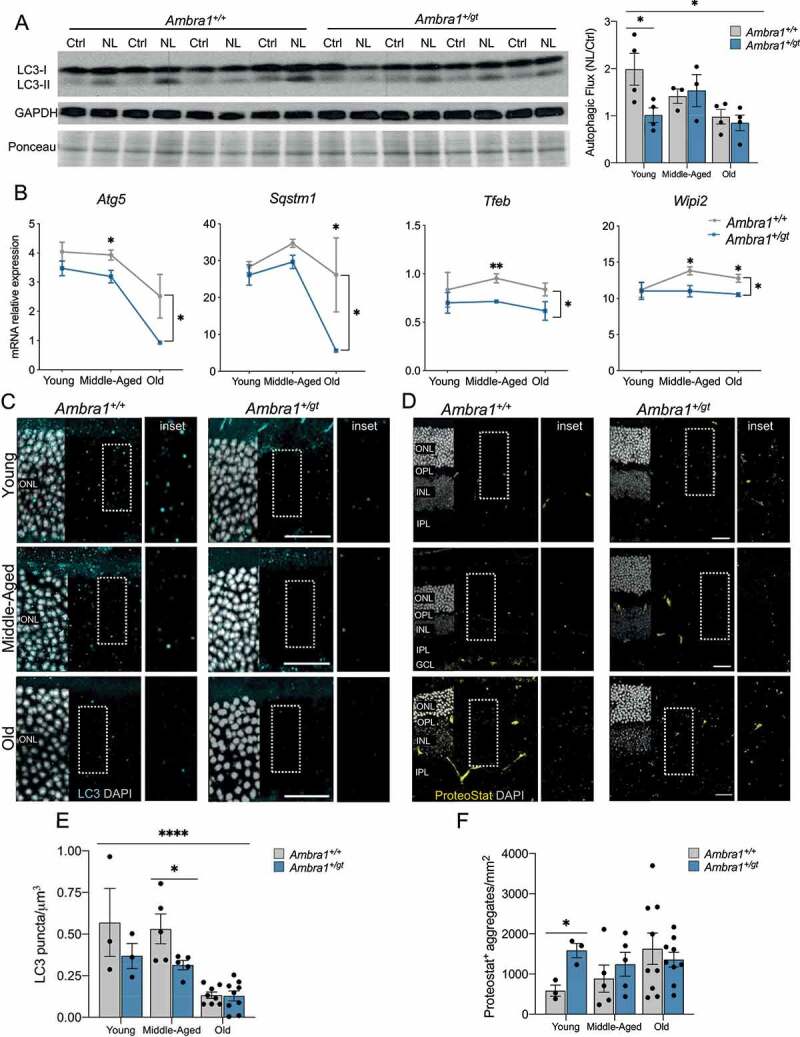
*Ambra1^+/gt^* retinas show a pronounced decrease in autophagy and increased protein aggregation. (A) Changes in autophagic flux determined by western blot for LC3-II in the absence or presence of NH_4_Cl and leupeptin (NL, to block lysosomal degradation) in whole retina extracts from young *Ambra1*^+/+^ and *Ambra1^+/gt^* mice. Quantification of autophagic flux in young (3 months), middle-aged (MA) (12–15 months), and old mice (22–26 months) is shown in the right-hand panel. Western blots corresponding to middle-aged and old mice are shown in Figure S1. Autophagic flux is determined as the ratio between NL and Control (n = 3–4 per group). (B) Decreased mRNA expression of *Atg5, Sqstm1, Tfeb* and *Wipi2* as determined by qPCR of whole retina extracts from young, middle-aged, and old *Ambra1*^+/+^ and *Ambra1*^+/gt^ mice (n = 3–4 per group). (C-F) Representative images and quantification of LC3 puncta in the outer nuclear layer (cyan) (C) and protein aggregates in the whole retina as measured by ProteoStat® protein aggregation assay (yellow) (D) in young, middle-aged, and old *Ambra1*^+/+^ and *Ambra1^+/gt^* mice. Nuclei were counterstained with DAPI (gray). Graphs show quantification of LC3^+^ puncta (E) and protein aggregates (F) (n = 3–9 per group). Data are presented as the mean ± SEM. *p < 0.05: two-tailed Student´s *t*-test or Mann-Whitney *U*-test (A, E, F); two-way ANOVA followed by *post hoc* LSD test for age (A and E) and genotype (**B**). Scale bars: 25 µm.

We investigated how a moderate reduction in autophagy, as occurs during physiological aging, impacts retinal homeostasis. We used *Ambra1^+/gt^* haploinsufficient mice [16], which are viable and display slight reductions in autophagic flux [25]. This outbred model, which has a CD1 background, provides a suitable means of studying age-dependent changes in a physiologically relevant context [26]. Autophagic flux decreased with age in this mouse strain, in agreement with our previous findings (Figures 1A and S1B) [10]. Retinas from *Ambra1^+/gt^* mice displayed reduced *Ambra1* gene expression (Figure S1A) and a parallel reduction in autophagic flux compared with control (*Ambra1^+/+^*) littermates at a young age (3–4 months old) (Figure 1A). Compared with control littermates, gene expression of many essential autophagy regulators such as *Atg5* and *Sqstm1* was already diminished in middle-aged (MA, 12–15 months) *Ambra1^+/gt^* mice, and further decreased in old (22–26 months) mice (Figure 1B). At the protein level, ATG12–ATG5 conjugation or unconjugated ATG5 were decreased slightly in parallel with the age-associated decline in autophagy. *Ambra1* haploinsufficiency was not associated with any alterations in Atg5 forms (Figure S1D). Moreover, *Ambra1^+/gt^* mice displayed a sustained reduction in mRNA expression of genes such as *Tfeb* and *Wipi2* (Figure 1B), suggesting exacerbation of autophagy deficiency in old *Ambra1^+/gt^* mice. Histological analysis revealed a progressive reduction of LC3^+^ puncta with increasing age in all retinal layers, an effect that was more pronounced in *Ambra1^+/gt^* mice (Figure 1C, E and S1E). In agreement with the observed reduction in autophagic flux in young animals, *Ambra1^+/gt^* mice showed a greater accumulation of protein aggregates as measured using the ProteoStat® protein aggregation assay (Figure 1D, F). These differences were abolished with increasing age: in control *Ambra1^+/+^* littermates protein aggregation increased with age. Together, these results demonstrate an age-associated decline in autophagy function that correlates with the loss of proteostasis, a phenotype already evident in young *Ambra1^+/gt^* mice.

**Figure 2.**
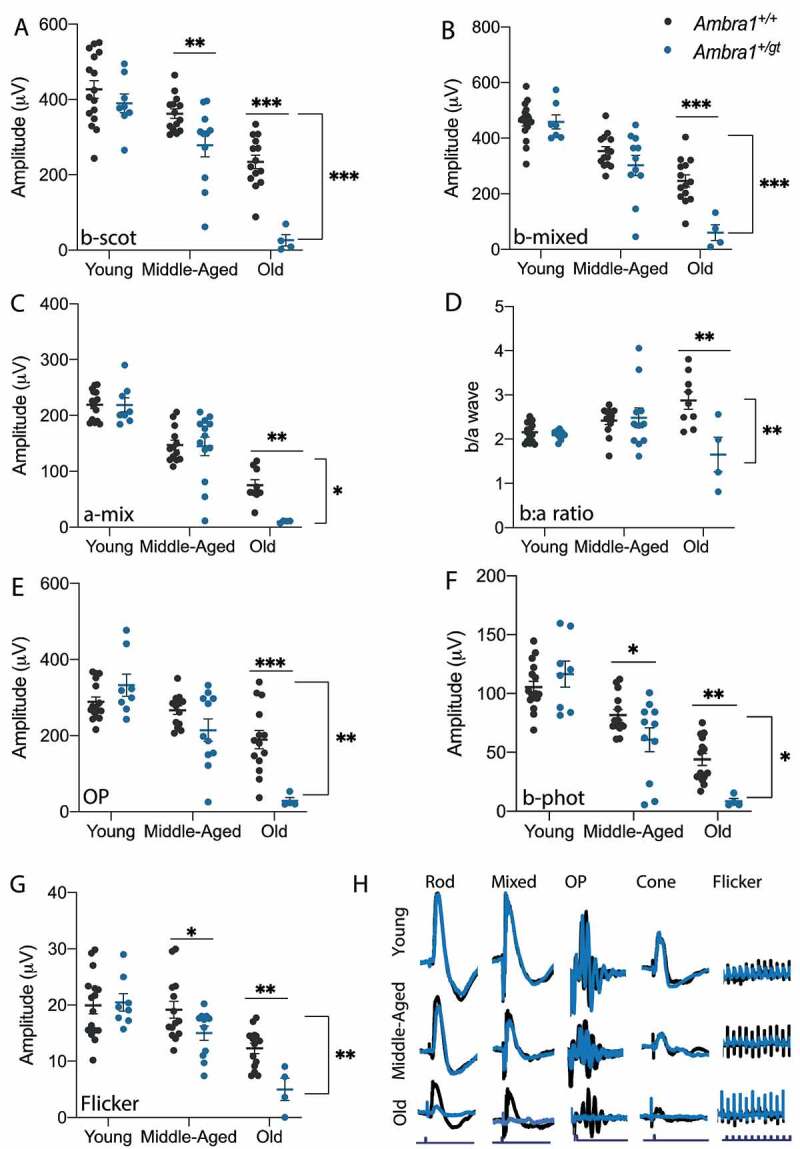
Age-dependent vision loss is exacerbated in *Ambra1^+/gt^* mice. Visual function assessed by measuring electroretinographic responses in young, middle-aged, and old *Ambra1*^+/+^ and *Ambra1^+/gt^* mice. (A) b-scotopic (b-Scot), (B) b-mixed (b-mix), and (C) a-mixed (a-mix) waves, and (D) b:a wave ratio and (E) oscillatory potentials (OP) were measured in dark-adapted conditions, and (F) b-photopic (b-phot) and (G) flicker waves were measured in light-adapted conditions. (H) Representative electroretinographic recording waves of young (n = 11–16), middle-aged (n = 8–14), and old (n = 4–14) *Ambra1*^+/+^ and *Ambra1^+/gt^* mice. Data are presented as the mean ± SEM. *p < 0.05, **p < 0.01, ***p < 0.001: two-way ANOVA (bracket bars) followed by Fischer’s LSD *post hoc* test for genotype (A–H).

Defective autophagy in the *Ambra1^+/gt^* retina was accompanied by reduced visual function, an effect that was already significant in middle-aged animals compared with control littermates (Figure 2A-H). In low light (scotopic) conditions, we observed decreased amplitudes of b-scot (low intensity stimulus) and both a- and b-mixed (high intensity stimulus) waves, implying decreased photoreceptor and bipolar cell signal transduction, respectively, in *Ambra1^+/gt^* versus *Ambra1^+/+^* mice (Figure 2A,B). In photopic conditions, decreased amplitudes of b-photopic and flicker waves indicated defective cone-dependent responses (Figure 2F,G). More detailed analysis based on b:a wave ratio showed a significant reduction in old *Ambra1^+/gt^* versus *Ambra1^+/+^* animals (Figure 2D), suggesting poor performance of bipolar cells at this age due to a more pronounced reduction of b-wave than a-wave amplitudes. Finally, diminished oscillatory potentials (OP) suggested poor performance of inner layer cells (Figure 2E). Regression analyses revealed that monoallelic deletion of *Ambra1* resulted in faster age-associated vision loss than in control mice, as evidenced by a significantly steeper slope for *Ambra1^+/gt^* versus *Ambra1^+/+^* mice for all parameters studied (Figure S2).

**Figure 3.**
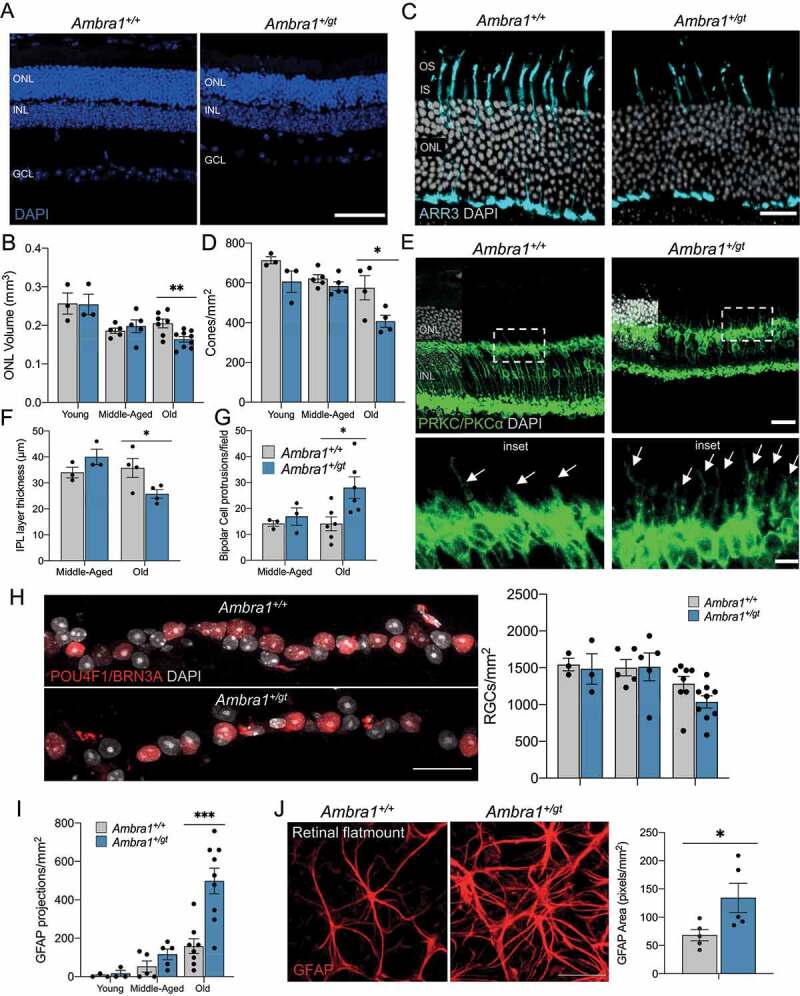
Retinal degeneration is observed in old *Ambra1^+/gt^* retinas. (A) Representative images of retinal nuclei (DAPI, blue). (B) Graph depicts changes in ONL volume in *Ambra1^+/+^* and *Ambra1^+/gt^* (n = 3–8 per group) mice over time. (C) Cone morphology evaluated using ARR3 immunostaining (cyan) and (D) corresponding quantification in young, middle-aged, and old *Ambra1^+/+^* and *Ambra1^+/gt^* mice (n = 3–5 per group). (E) Immunostaining of bipolar cells (PRKCA/PKCα, green) in old *Ambra1^+/+^* and *Ambra1^+/gt^* mice. Insets (below) show protrusions from bipolar cells toward the outer nuclear layer (ONL) (arrows). (F) Quantification of inner plexiform layer (IPL) thickness (n = 3–4 per group). (G) Determination of number of protrusions from bipolar cells (n = 3–4 per group). (H) Immunostaining of retinal ganglion cells with POU4F1/BRN3A (red) and corresponding quantification in young, middle-aged, and old *Ambra1*^+/+^ and *Ambra1*^+/gt^ retinas (n = 3–8). (I) Quantification of GFAP-positive projections toward the outer retina in each experimental group (n = 3–9). (J) GFAP immunostaining (red) of retinal flat mounts from old *Ambra1^+/+^* and *Ambra1^+/gt^* mice and quantification of GFAP-positive area (n = 5 per group). Data are presented as the mean ± SEM. *p < 0.05, **p < 0.01, ***p < 0.001: two-tailed Student’s *t*-test (**B, D, F, G, H, I** and **J**). Scale bars: 50 µm (A and C); 25 µm (**C, H** and **J**); 10 µm (insets in **E**).

To examine the effects of the age-associated decline in autophagy on retinal morphology, we compared ONL (outer nuclear layer) nuclear density in young, middle-aged, and old *Ambra1^+/gt^* mice versus *Ambra1^+/+^* littermates. Old *Ambra1^+/gt^* mice showed reduced ONL volume density (Figure 3A,B), but no significant increase in the number of apoptotic TUNEL-positive cells (Figure S3A). These apoptotic cells may have been already removed by the more numerous microglial cells with amoeboid morphology, often associated to their phagocytic phenotype (Figure S3C). The number of cones stained with either ARR3/Cone Arrestin (arrestin 3, retinal) or OPN1MW/red/green opsin (opsin 1 (cone pigments), medium-wave-sensitive (color blindness, deutan)) was reduced in aged *Ambra1^+/gt^* retinas (Figure 3C,D and S3B), indicating that *Ambra1* haploinsufficiency results in an age-associated reduction in cone population. We next investigated whether monoallelic loss of *Ambra1* results in alterations in the inner retina. *Ambra1^+/gt^* mice showed a reduction in the thickness of the inner plexiform layer (IPL) (Figure 3E,F) compared with *Ambra1^+/+^* littermates, suggesting reduced synapsis between retinal neurons. Moreover, in these mice, bipolar cells showed protrusions toward the ONL (Figure 3E [insets], G), a phenotype associated with the bipolar cell response to photoreceptor retraction and connectivity loss [27]. In terms of photoreceptor and bipolar morphology and connectivity, the observed phenotypes match perfectly with the loss in visual function revealed by electroretinogram. Old *Ambra1^+/gt^* and control *Ambra1^+/+^* mice showed no significant differences in the number of retinal ganglion cells stained with the specific transcription factor POU4F1/BRN3A (Figure 3H).

Müller cells are retinal glial cells that provide trophic factors and support the neuroretina. In response to damage stimuli, Müller cells activate and extend GFAP-positive projections toward the outer retina. A persistent inflammatory and gliotic response accompanying aging has been described in mice [28]. Although we did not observe major morphological changes in Müller glia stained with the Müller cell marker GLUL (glutamate-ammonia ligase (glutamine synthetase)) (Figure S3D), we observed a massive increase in the number of GFAP projections toward the ONL in aged *Ambra1^+/gt^* versus *Ambra1^+/+^* mice (Figure 3I). This phenotype was accompanied by a larger area of GFAP^+^ staining at the level of the ganglion cell layer in old *Ambra1^+/gt^* retinal flat mounts (Figure 3J), indicating astrocyte hypertrophy. In agreement with this gliosis response, we observed a slight increase in amoeboid microglia in old *Ambra1^+/gt^* versus *Ambra1^+/+^* retinas (Figure S3C) that could be linked to their activated state during inflammation [29,30]. Taken together, these data suggest that *Ambra1* haploinsufficiency results in an accelerated retinal aging phenotype, accompanied by a proinflammatory state and vision defects.

### Monoallelic deletion of *Ambra1* results in metabolic alterations in the retina associated with mitochondrial dysfunction

**Figure 4.**
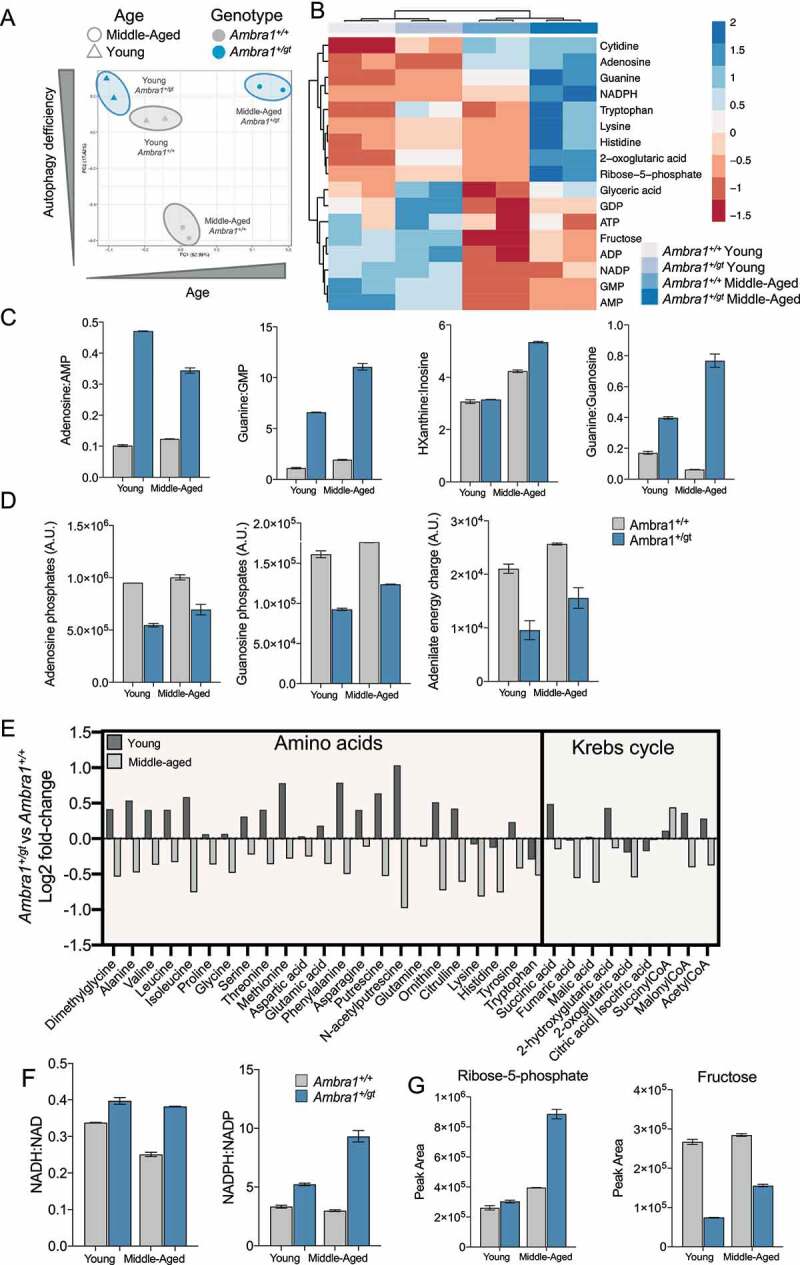
Metabolic imbalance in *Ambra1^+/gt^* mice. (A) Principal component analysis (PCA) of retinal mentabolites in samples from young and middle-aged *Ambra1^+/+^* and *Ambra1^+/gt^* retinal metabolites. (B) Heatmap and hierarchical clustering (HCL) analysis of metabolites for which statistically significant alterations were observed in *Ambra1^+/gt^* mice (n = 2 pools of 4 retinas per group). Statistical significance (Skillings-Mack test) was set at p < 0.05. (C) Purine metabolism metabolites as determined by the ratios of related metabolic compounds in young and middle-aged *Ambra1^+/+^* and *Ambra1^+/gt^* retinas (n = 2 per group). (D) Pools of charged purine (adenosine and guanosine) phosphate nucleotides and estimation of the adenylate energy charge in young and middle-aged *Ambra1^+/+^* and *Ambra1^+/gt^* retinas (n = 2 per group). (E) Fold changes in the levels of amino acids and Krebs cycle-related metabolites in young and middle-aged *Ambra1^+/+^* versus *Ambra1^+/gt^* mice (n = 2 per group). (F) Retinal redox status as measured by the ratios of NAD-related metabolites in young and middle-aged *Ambra1^+/+^* versus *Ambra1^+/gt^* littermates (n = 2 per group). (G) Changes in the pentose-phosphate pathway as determined based on relative levels of representative metabolites (fructose and ribose-5-phosphate) in young and middle-aged *Ambra1^+/+^* versus *Ambra1^+/gt^* littermates (n = 2 per group). Data are presented as the mean ± SEM.etc.].

Recent findings by our group demonstrate that autophagy-deficient animals display marked metabolic alterations in the embryonic retina [31], and that retinal degeneration is often associated with metabolic imbalances [32]. We next explored how deficient autophagy influences the retinal metabolome in the early stages of degeneration by comparing retinas from young and middle-aged *Ambra1^+/+^* and *Ambra1^+/gt^* mice. Metabolite levels were determined by liquid chromatography/gas chromatography–mass spectrometry (LC/GC–MS) and concentrations represented as the area under the curve and metabolite ratios. Although sample correlation, principal component analysis, and unsupervised hierarchical clustering analysis revealed greater differences for age than for genotype (Figure 4A, and S4A-B), middle-aged *Ambra1^+/gt^* retinas showed the most pronounced changes in retinal metabolome.

To determine which metabolites were dysregulated, we performed a Skillings-Mack analysis to identify significant genotype-related effects, followed by an enrichment analysis. Of the 100 metabolites measured (Figure S4A), 16 were significantly altered in middle-aged *Ambra1^+/gt^* retinas (Figure 4B). Those metabolites are implicated in purine metabolism, the pentose phosphate pathway, and the Warburg effect, among other processes (Figure S4C). Nucleotide metabolite ratios revealed alterations in adenine and guanine metabolism in *Ambra1^+/gt^* versus *Ambra1^+/+^* retinas (Figure 4C). Furthermore, middle-aged *Ambra1^+/gt^* retinas displayed marked reductions in total nucleotide phosphate levels and adenylate energy charge (Figure 4D), a phenotype associated with energetic underperformance [33]. Interestingly, while amino acid levels were increased in young *Ambra1^+/gt^* versus *Ambra1*^+/+^ mice, levels of most amino acids analyzed and of many TCA cycle intermediates were reduced in middle-aged *Ambra1^+/gt^* mice (Figure 4E). Finally, the NADH:NAD and the NADPH:NADP ratios were altered and levels of ribose-5-phosphate increased in middle-aged *Ambra1^+/gt^* versus *Ambra1*^+/+^ mice (Figure 4F-G), indicating marked metabolic alterations suggestive of defective oxidative metabolism in *Ambra1^+/gt^* mice.

**Figure 5.**
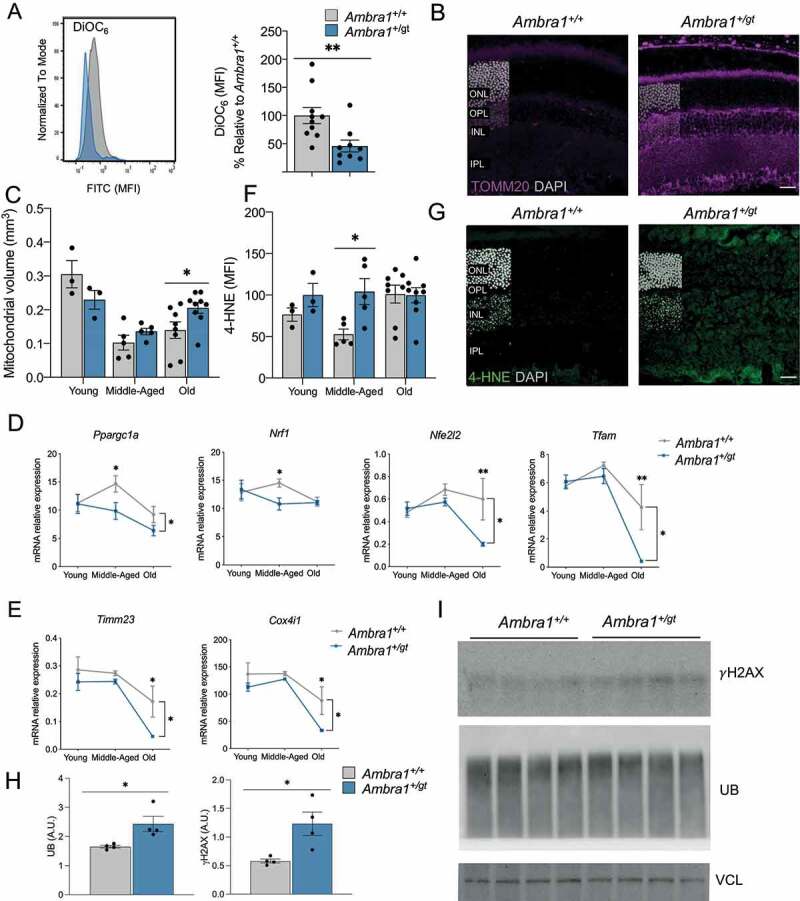
Mitochondrial alterations, cellular damage, and oxidative stress in *Ambra1^+/gt^* retinas. (A) Fluorescence-activated cell sorting (FACS) histogram (left) and quantification (right) of mitochondrial membrane potential measured by DiOC6(3) probe in dissociated retinas from young *Ambra1^+/+^* and *Ambra1^+/gt^* mice (n = 9–10 per group). (B-C) Immunostaining of mitochondrial mass (TOMM20, magenta). (B) Representative image of old *Ambra1^+/+^* and *Ambra1^+/gt^* retinas and (C) quantification of mitochondrial volume in young, middle-aged, and old *Ambra1^+/+^* and *Ambra1^+/gt^* retinas (n = 3–8 per group). (D–E) Mitochondrial transcriptional signature is decreased in *Ambra1^+/gt^* retinas. Graphs depict the transcriptional levels of mitochondrial biogenesis related-genes (*Ppargc1a, Nrf1, Nfe2l2*, and *Tfam*) (D) and mitochondrial mass-related genes (*Timm23* and *Cox4i1*) (E). (F-G) Immunostaining to determine lipid peroxidation levels (4-HNE, green) in middle-aged and *Ambra1^+/+^* and *Ambra1^+/gt^* retinas and subsequent quantification of 4-HNE mean fluorescence intensity (MFI) in the ONL of young, middle-aged, and old *Ambra1^+/+^* and *Ambra1^+/gt^* littermates (n = 3–8 per group). Nuclei are counterstained with DAPI (gray). (H-I) Protein levels of γ-H2AX, UB/ubiquitin, and VCL/vinculin (loading control) in middle-aged *Ambra1^+/+^* and *Ambra1^+/gt^* retinas as determined by western blot and quantification of protein levels is shown on the right (n = 4 per genotype). Data are presented as the mean ± SEM. *p < 0.05, **p < 0.01: two-tailed Student´s *t*-test or Mann-Whitney *U*-test (**A, C, F** and **H**) and two-way ANOVA followed by Fisher’s LSD *post hoc* test for genotype (D, E). Scale bars: 25 µm.

We next investigated whether alterations in mitochondrial function in *Ambra1*-deficient retinas could explain the exacerbated metabolic dysregulation observed in middle-aged animals. Surprisingly, marked reductions in mitochondrial membrane potential as determined by flow cytometry with DiOC_6_ were already detectable in young *Ambra1^+/gt^* retinas (Figure 5A). TOMM20 immunostaining revealed augmented mitochondrial mass in most retinal cell layers in aged *Ambra1^+/gt^* animals (Figure 5B,C). Increases in mitochondrial biogenesis can often compensate for mitochondrial alterations. However, *Ambra1^+/gt^* animals displayed no such changes and old animals even showed reduced mRNA expression of the main mitochondrial biogenesis regulators *Ppargc1a, Nrf1, Nfe2l2*, and *Tfam* (Figure 5D), as well as decreased expression of mitochondrial genes such as *Timm23* and *Cox4i1* (Figure 5E). Thus, the increased mitochondrial mass in the absence of biogenesis suggests a defect in the removal of dysfunctional mitochondria via mitophagy in aged *Ambra1^+/gt^* animals.

We further investigated whether these mitochondrial alterations result in oxidative damage to intracellular membranes. Photoreceptor outer segments (POS) are enriched in lipids that are susceptible to peroxidation upon oxidative stress, affecting their functionality [34]. Compared with control littermates, middle-aged *Ambra1^+/gt^* mice showed increased levels of lipid peroxidation in all retinal layers, as determined by measuring 4-hydroxynonenal (4-HNE) (Figure 5F,G), and other signs of cellular damage such as increased γH2AX, and ubiquitin levels, indicative of DNA damage and decreased proteasome activity, respectively (Figure 5H,I). Together, these data indicate marked mitochondrial alterations in *Ambra1*-deficient retinas, including decreased mitochondrial membrane potential in young animals that cannot be compensated for in later life by autophagy or mitochondrial biogenesis, potentially resulting in lipid peroxidation and oxidative damage to cellular membranes.

**Figure 6.**
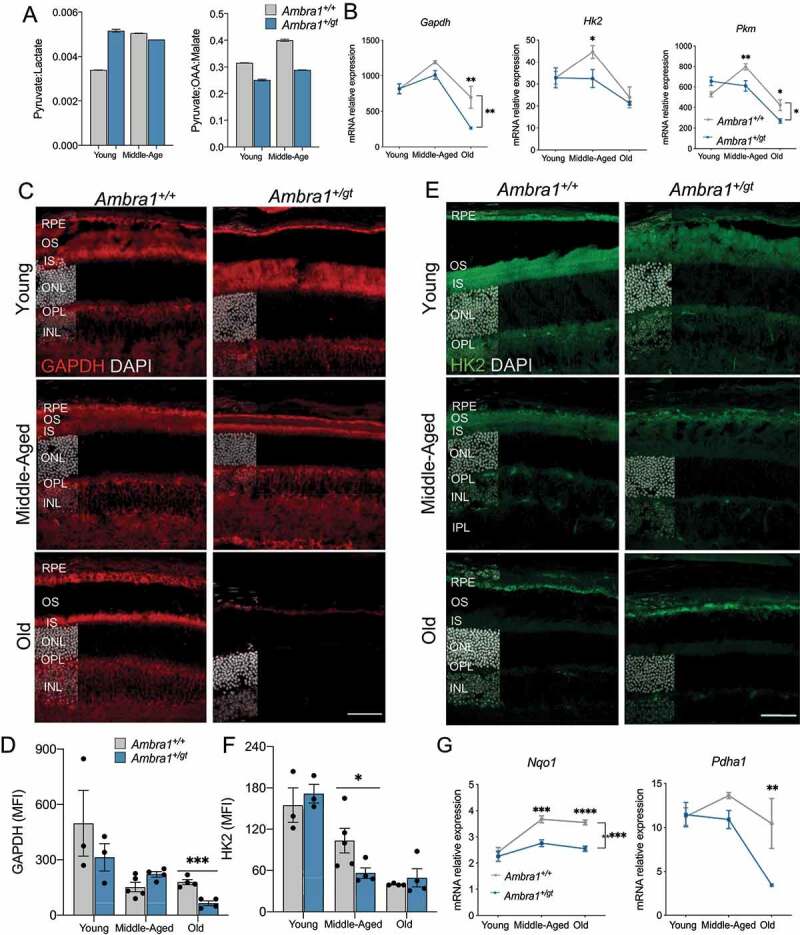
Glycolytic disfunction in *Ambra1^+/gt^* retinas. (A) Retinal metabolic status as determined by ratios of glycolytic-end metabolites in young and middle-aged *Ambra1^+/+^* and *Ambra1^+/gt^* mice (n = 2 per group). (B) mRNA expression of glycolysis-related genes (*Gapdh, Hk2*, and *Pkm)* in young, middle-aged, and old *Ambra1^+/+^* and *Ambra1^+/gt^* retinas. (C) Immunostaining for GAPDH (red) in young, middle-aged, and old *Ambra1^+/+^* and *Ambra1^+/gt^* retinas (n = 3–4 per group) and (D) corresponding quantification. (E) Immunostaining for HK2 (green) in young, middle-aged, and old *Ambra1^+/+^* and *Ambra1^+/gt^* retinas (n = 3–4 per group) and (F) corresponding quantification. Nuclei in **C** and **E** are counterstained with DAPI (gray). (G) mRNA expression of redox status-related enzymes (*Nqo1 and Pdha1)* as determined by qPCR of whole retina extracts from young, middle-aged, and old *Ambra1^+/+^* and *Ambra1^+/gt^* mice (n = 3–4 per group). Data are presented as the mean ± SEM. *p < 0.05, **p < 0.01, ***p < 0.001, ****p < 0.0001: two-tailed Student´s *t*-test (D and F) and two-way ANOVA (bracket bars) followed by Fisher’s LSD *post hoc* test for genotype (B and G). Scale bars in **E** and **F**: 50 µm.

Our enrichment analysis (Figure S4C) revealed upregulation of the pathway underlying the Warburg effect. Because the pyruvate:lactate ratio was increased in young *Ambra1^+/gt^* versus *Ambra1*^+/+^ mice (Figure 6A), we next examined mRNA expression of the main glycolytic enzymes. In all age groups, *Ambra1^+/gt^* retinas showed reduced mRNA expression of many glycolytic enzymes, including *Gapdh, Hk2*, and *Pkm* (Figure 6B), and a tendency toward reduced GAPDH and HK2 immunofluorescence (Figure 6C-F). In agreement with our metabolic findings in middle-aged *Ambra1^+/gt^* retinas, we observed decreased transcription of enzymes that help maintain cellular redox status. For example, we observed reduced expression of *Nqo1* (NAD(P)H dehydrogenase, quinone 1) (Figure 6G), an enzyme that oxidizes NADH to NAD in the electron transport chain, in agreement with the aforementioned alterations in NADH:NAD ratio (Figure 4F). Furthermore, we detected lower mRNA levels of *Pdha1* (pyruvate dehydrogenase E1subunit alpha 1). PDHA1 protein is crucial to allow entry of acetyl-CoA produced from pyruvate into the TCA cycle, where it serves as a source of carbon for anabolism (Figure 6G). Together these data demonstrate that mitochondrial alterations already evident in young *Ambra1^+/gt^* retinas may underlie the metabolic changes, including decreased glycolysis and energetic underperformance, observed in later life.

## *RPE alterations in* Ambra1^+/gt^ *mice result in increased susceptibility to stress*

**Figure 7.**
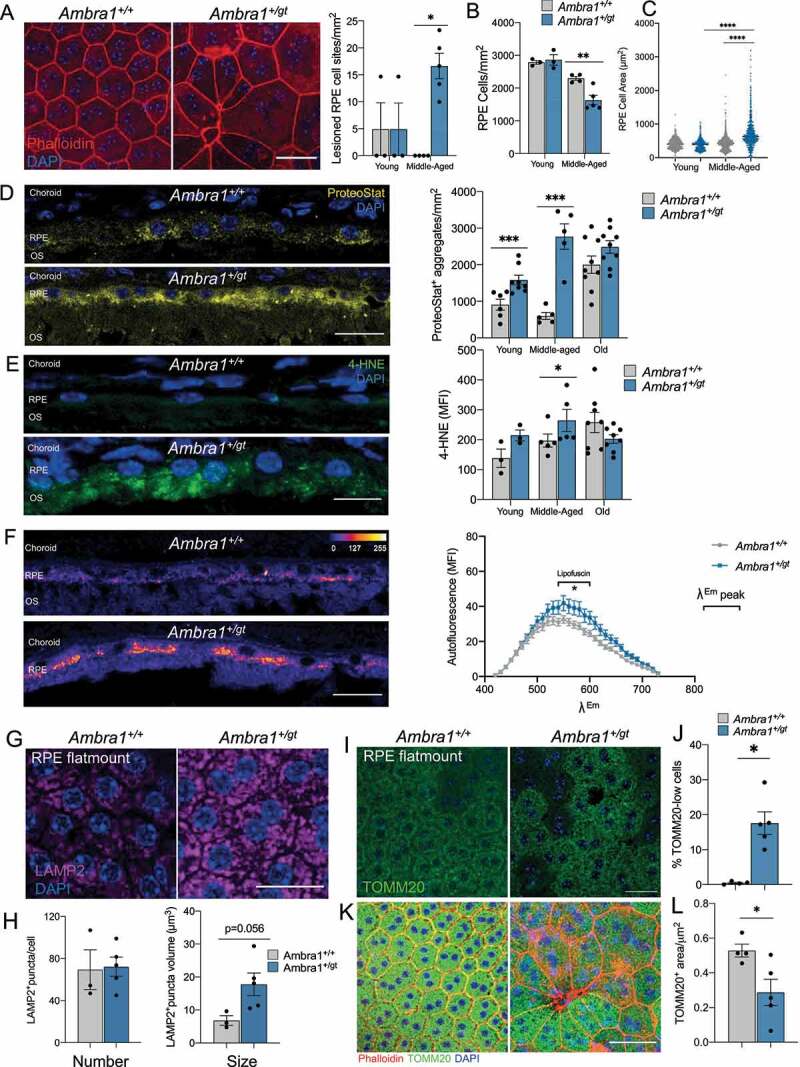
*Ambra1^+/gt^* mice display abnormalities in the retinal pigment epithelium (RPE) associated with premature aging. (A) RPE morphology assessed by phalloidin (red) staining in RPE flat mounts from middle-aged *Ambra1^+/+^* and *Ambra1^+/gt^* littermates, showing increases in cell size and lesion site number in the RPE monolayer in *Ambra1^+/gt^* mice (left). Graph on the right shows the quantification (right) of lesion site number (dying/dead RPE cells; points where >3 cells are in contact) per mm^2^ (n = 3–5 per group). (B) Quantification of the number of cells/mm^2^ in RPE flat mounts from young and middle-aged *Ambra1^+/+^* and *Ambra1^+/gt^* littermates (n = 3–5 per group). (C) Quantification of the area of individual RPE cells (µm^2^) in RPE flat mounts from young and middle-aged *Ambra1^+/+^* and *Ambra1^+/gt^* littermates (n = 3–5 animals per genotype/age group, >350 cells per group). (D) ProteoStat® assay to detect protein aggregation (yellow), showing the accumulation of protein aggregates in the RPE of young, middle-aged, and old *Ambra1^+/+^* and *Ambra1^+/gt^* littermates. Single z-images are shown. Right-hand panel shows quantification of protein aggregates (n = 5–8 per group). (E) Lipid peroxidation as determined by 4-HNE staining (green) in young, middle-aged, and old *Ambra1^+/+^* and *Ambra1^+/gt^* littermates and corresponding quantification of mean fluorescence intensity (MFI) (n = 3–5 per group). (F) Measurement of autofluorescence in the RPE using Lambda-scan to capture the entire emission spectrum upon UV excitation in old *Ambra1^+/+^* and *Ambra1^+/gt^* littermates (n = 5–8 per group). (G) RPE flat mounts from middle-aged *Ambra1^+/+^* and *Ambra1^+/gt^* immunostained with the lysosomal membrane marker LAMP2 (nuclei are counterstained with DAPI [blue]), and (H) corresponding quantification of the number and individual volume of puncta (n = 3–5 per group). (I–K) Immunostaining of mitochondria (TOMM20, green) in RPE flat mounts from middle-aged *Ambra1^+/+^* and *Ambra1^+/gt^* littermates. Nuclei were counterstained with DAPI (blue) and the F-actin marker phalloidin (red). (J–L) Quantification of TOMM20 staining in **I–K**. Data are presented as the mean ± SEM. *p < 0.05, **p < 0.01; ***p < 0.001; ****p < 0.0001: two-tailed Student’s *t*-test (**B, D–F**), Mann-Whitney *U*-test (**A, H, J** and **L**), or Kruskal-Wallis test followed by Dunn’s *post hoc* test (**C**). Scale bars: 25 µm.

Retinal metabolism relies heavily on the RPE, and vice versa. For example, photoreceptors mainly obtain energy through glycolysis from glucose provided from the outer retinal blood supply and transported through the RPE, and, in exchange, return lactate and lipid-containing outer segments that are used to fuel oxidative phosphorylation in the RPE [35]. Because alterations in RPE homeostasis have important consequences for retinal metabolism and function, we examined age-associated changes in RPE function in *Ambra1^+/gt^* mice. While the RPE in young *Ambra1^+/gt^* mice showed no alterations with respect to control littermates, phalloidin staining in middle-aged *Ambra1^+/gt^* animals revealed important RPE lesions corresponding to monolayer disruption (Figure 7A) [36]. Moreover, middle-aged *Ambra1^+/gt^* mice displayed fewer (Figure 7B) and larger (Figure 7C) RPE cells, phenotypes associated with RPE cell death, whereby the remaining cells expand to cover the area left by dying cells [36]. ProteoStat protein aggregation assay showed that monoallelic loss of *Ambra1* was associated with increased proteotoxicity, an effect that was already significant in young animals (Figure 7D). Importantly, those differences were no longer observed in old animals, as protein aggregation was also increased in the old control animals. In agreement, autophagosome number determined by LC3 staining was reduced both in flat mounts and RPE cryosections from *Ambra1^+/gt^* versus *Ambra1^+/+^* mice (Figure S5A-D). The age-associated increase in lipid peroxidation was significantly augmented in the RPE of middle-aged *Ambra1^+/gt^* mice (Figure 7E). Chronic RPE dysfunction is associated with the appearance of autofluorescent lipofuscin aggregates [34]. Lambda-scan autofluorescence analysis showed specific accumulation of lipofuscin inside swollen lysosomes in old *Ambra1^+/gt^* mice (Figure 7F). In agreement, lysosomes in *Ambra1^+/gt^* animals were similar in number to those of control littermates, but with greater individual volume (Figure 7G,H). In summary, these data show that monoallelic loss of *Ambra1* is associated with marked alterations in the RPE from a young age. These changes include a reduction in autophagy that was paralleled by increased protein aggregation, oxidative damage, lysosomal alterations, and lipofuscin accumulation, resulting in RPE cell death by middle age.

As previously stated, mitochondrial function is required to sustain ATP synthesis in the RPE. RPE flat mounts from middle-aged *Ambra1^+/gt^* animals displayed numerous cells with markedly reduced mitochondria mass, as determined by TOMM20 staining (Figure 7I,J). Those mitochondria-poor cells were enlarged and showed alterations in cytoskeleton architecture, as assessed by phalloidin staining (Figure 7K,L), suggesting a correlation between reduced mitochondrial mass and RPE hypertrophy. Western blot for TOMM20 and TIMM23 also revealed a trend toward reduced mitochondria protein levels (Figure S5E). Given the high degree of metabolic interdependence between the retina and RPE, we hypothesize that alterations in mitochondrial function may make the RPE much more dependent on glucose for sustained ATP synthesis. In agreement, our data show a tendency toward increased SLC2A1/GLUT1 glucose transporter expression in the RPE of *Ambra1^+/gt^* animals, and a tendency toward increased glycolytic GAPDH expression in the RPE of young and middle-aged *Ambra1^+/gt^* mice (Figure S5F, G), pointing to increased glucose demand in the RPE. Finally, the accumulation of activated phagocytic cells was evidenced by an increase in the number of amoeboid cells positive for AIF1/IBA1 (allograft inflammatory factor 1) staining in the RPE in middle-aged *Ambra1^+/gt^* mice (Figure S5H). Taken together, these data indicate that the autophagy defect with *Ambra1* haploinsufficiency results in reduced mitochondrial mass, and metabolic reprogramming that favors glycolysis to fuel ATP synthesis.

**Figure 8.**
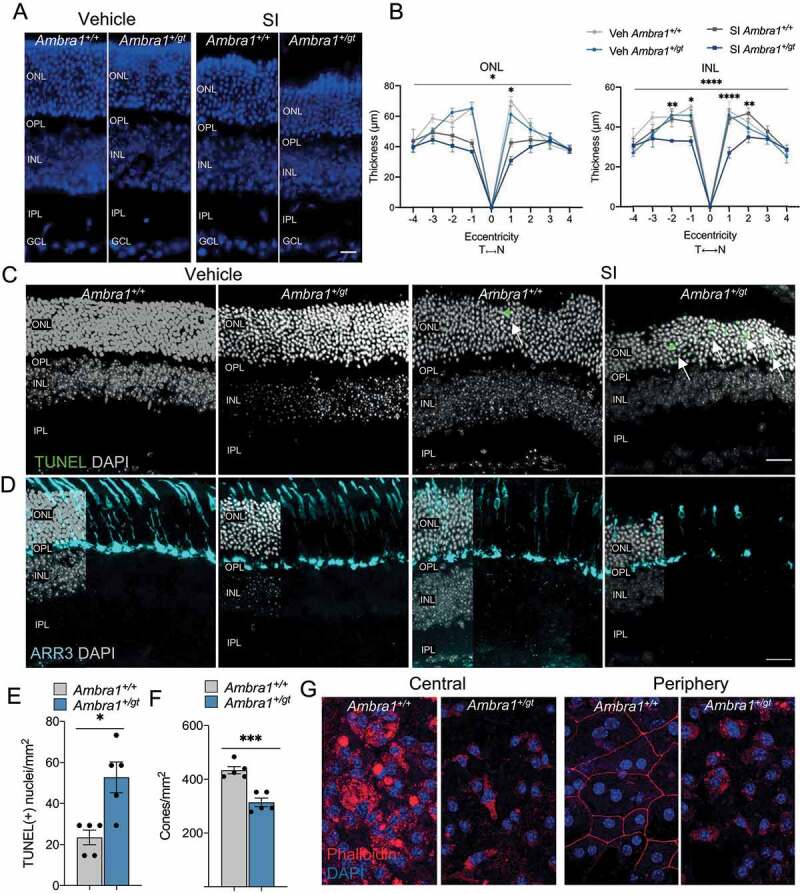
Increased sensitivity to sodium iodate in the retina and RPE of *Ambra1^+/gt^* mice. (A) Representative DAPI-stained retinal cryosections from middle-aged *Ambra1^+/+^* and *Ambra1^+/gt^* littermates injected with either 50 mg/kg sodium iodate (SI) or vehicle (PBS) and analyzed after 7 days. (B) Quantification of the thickness of the outer nuclear layer (ONL) and inner nuclear layer (INL) of middle-aged *Ambra1^+/+^* and *Ambra1^+/gt^* littermates treated with SI or vehicle (n = 4–5 per group). (C- D) Staining of apoptotic cells with TUNEL (**C**, green) and cones (ARR3; **D**, cyan) in retinal cryosections from middle-aged *Ambra1^+/+^* and *Ambra1^+/gt^* littermates injected with SI or vehicle. Nuclei were counterstained with DAPI (gray). Single z-images were analyzed (E) Quantification of the number of TUNEL-positive photoreceptor nuclei in middle-aged *Ambra1^+/+^* and *Ambra1^+/gt^* littermates treated as in C (n = 5 per group). (F) Quantification of the number of cone photoreceptors of middle-aged *Ambra1^+/+^* and *Ambra1^+/gt^* littermates treated as in **D** (n = 5 per group). (G) Phalloidin (red) and DAPI (blue) immunostaining of RPE flat mounts (images from central and peripheral areas are shown) from middle-aged *Ambra1^+/+^* and *Ambra1^+/gt^* littermates injected with 50 mg/kg SI or vehicle. Data are presented as the mean ± SEM. *p < 0.05, ***p < 0.001, ****p < 0.0001: two-way ANOVA followed by Fisher’s LSD *post hoc* test for genotype (**B**); unpaired Student’s *t* test (D); or Mann-Whitney *U-*test (**E**). Scale bars: 15 µm (**A**); 50 µm (C and D).

In age-related diseases there is usually an interplay between physiological aging and both genetic and environmental factors. Since the alterations in the RPE of *Ambra1^+/gt^* mice occur earlier and are more severe than those seen in the neuroretina, we used a pharmacological model of RPE degeneration to assess vulnerability to external stressors and age-related defects in these mice [37,38]. Middle-aged mice were treated with sodium iodate (SI), a well-studied model of primary RPE damage that leads to secondary retinal degeneration. Middle-aged *Ambra1^+/gt^* mice and control littermates were intraperitoneally injected with 50 mg/kg SI or vehicle and sacrificed 1 week later. No differences in retinal morphology or retinal layer thickness were observed in the vehicle-treated groups (Figure 8A-B). However, compared with control littermates, *Ambra1^+/gt^* mice were significantly more sensitive to SI-induced damage, as evidenced by a decrease in ONL thickness (Figure 8B). Surprisingly, SI-treated *Ambra1^+/gt^* mice also displayed a significant decrease in inner nuclear layer (INL) thickness (Figure 8B) that was not observed in SI-treated control littermates. This suggests an increased vulnerability of both retinal photoreceptors and interneurons to stress, correlating with loss of visual function in old animals. Supporting increased stress vulnerability in middle-aged *Ambra1^+/gt^* mice, these animals showed increased photoreceptor apoptotic cell death, as determined by TUNEL staining (Figure 8C,E), and decreased cone number after SI-induced damage (Figure 8D,F). SI treatment exerts a degenerative effect following a central to peripheral gradient [37]. Assessment of RPE monolayer morphology using phalloidin staining showed severe RPE disruption, even in the peripheral region of the retina, in SI-treated *Ambra1^+/gt^* mice (Figure 8G). Given the retinal phenotype observed in *Ambra1^+/gt^* mice, and the fact that SI exerts a time- and region-dependent effect [38], this finding demonstrates increased susceptibility of the *Ambra1^+/gt^* RPE to stress, resulting in exacerbated retinal degeneration.

### *Ambra1* is upregulated in RPE cells after SI treatment and in the macular region of age-related macular degeneration (AMD) patients

ARPE-19 cells are an RPE-derived human cell line [39] that has been widely used to study the effects of SI *in vitro*. Treatment of ARPE-19 cells with SI for 24 h resulted in loss of cell viability and mitochondria membrane potential, decreased mitochondrial mass, and increased lipid peroxidation (Figure S6A, B), characteristics that are phenotypically similar to that observed in the RPE of old *Ambra1^+/gt^* mice. Next, we analyzed a dataset derived from transcriptionally-profiled ARPE-19 cells treated for 24 h with SI (GSE142591) [40], and found that mRNA expression of *AMBRA1* as well as many genes of the autophagy-initiation complex, including *BECN1, ULK1*, and *RB1CC1*, were significantly upregulated in response to SI-induced damage (Figure S6C, D). Minor increases in mRNA levels were observed for autophagy core genes such as *MAP1LC3B, ATG5, ATG7*, and *ATG16L1* (Figure S6D). These results suggest upregulation of autophagy machinery is a mechanism to protect against SI-induced damage in RPE cells. Consistent with this idea, we also observe an increase in AMBRA1 expression in the retina of SI treated mice (Figure S6E). Together, these data indicate that *Ambra1-*associated autophagy in RPE may play a key protective role in preventing age- or stress-dependent RPE dysfunction.

**Figure 9.**
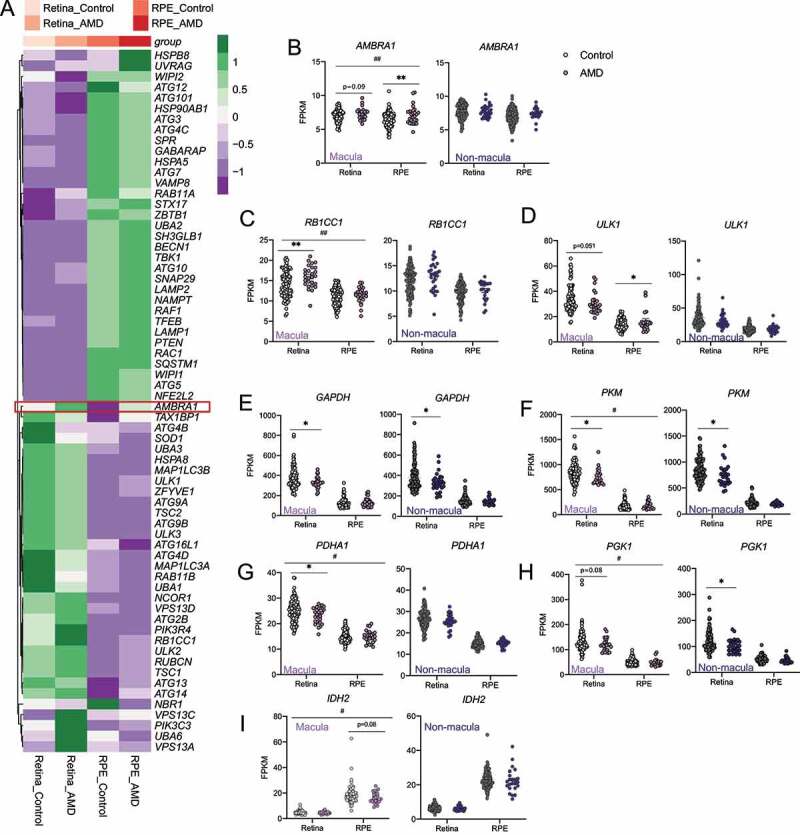
AMD is associated with upregulation of genes of the autophagy initiation machinery and downregulation of glycolysis-related genes. (A) Heatmap showing expression analysis of a manually-curated autophagy gene list in the macular region of the retina and RPE of control and AMD patient samples from the GSE135092 [47], dataset (n = 26–105). (B) Detailed expression analysis of *AMBRA1* from the aforementioned dataset in macular and non-macular regions (n = 26–105). (C–J) Detailed expression analysis of autophagy-related genes: *RB1CC1* (C), *ULK1* (D), *GAPDH* (E), *PKM* (F), *PDHA1* (G), *PGK1* (H), *IDH2* (J) in the aforementioned dataset in in macular and non-macular regions (n = 26–105). Data are presented as the mean ± SEM. *p < 0.05, **p < 0.01: two-tailed Student’s *t*-test (B–J). ^#^p < 0.05, ^##^p < 0.01: two-way ANOVA followed by Fisher’s LSD *post hoc* test for disease (**B–C, F–J**).

As defects in autophagy have been implicated in AMD pathology [41–43], we sought to determine what characteristics of our animal model with autophagy deficiency replicate features of AMD. Our data shown previously indicate that *Ambra1* haploinsufficiency results in RPE hypertrophy (Figure 7A-C) [36,44], increased oxidative stress (Figures 5F-G and 7E) [45], and inflammation (Figure 3I-J, S3C-D), features previously linked to AMD [46]. Using a publicly available dataset of RNA sequencing (RNA-seq) profiles of macular and non-macular regions of the human retina and RPE from healthy controls and AMD patients (GSE135092) [47], we investigated the expression of the main autophagy regulators (Figure 9A). While *AMBRA1* mRNA expression was increased in the macular region of AMD donors, no changes were observed in the non-macular region (Figure 9B). A similar trend was observed for *RB1CC1* and *ULK1* (Figure 9C). This gene expression pattern demonstrates that in AMD the autophagy initiation machinery is specifically induced in the macular region, but not in the non-macular region, and that this may constitute an important mechanism to combat the disease.

Metabolic underperformance in the RPE-retina induced by *Ambra1* deficiency was associated with RPE degeneration (Figure 7A,B) and loss of visual function (Figure 2). We therefore investigated whether the aforementioned RNA-seq dataset [47] revealed metabolic alterations in the retina and RPE in AMD patients. In line with our findings in *Ambra1*-deficient retinas, mRNA expression of many glycolytic enzymes was significantly decreased in both macular and non-macular regions of the retina from AMD patients. However, no glycolytic changes were observed in the RPE (Figure 9E-H). These observations, together with the observed decrease in RPE mRNA expression of IDH2 (Figure 9J), the limiting enzyme in the reductive carboxylation pathway essential for RPE metabolism, suggest decreased mitochondrial oxidative function in the RPE in AMD patients. Taken together, these findings support the view that defective mitochondrial function in the RPE results in a glucose metabolism imbalance, leading to decreased glycolysis in the retina. Moreover, the transcriptional upregulation of autophagy machinery, including *AMBRA1*, observed in the affected macular region in AMD patients suggests a key role of Ambra1-dependent autophagy in protecting the retina in AMD patients.

Taken together, these data demonstrate that *Ambra1* deficiency alters the retina-RPE ecosystem leading to RPE cell death, metabolic underperformance, loss of visual function, and increased susceptibility to stress. Our data also show that *Ambra1* expression is selectively upregulated in the macular, but not the non-macular, region of the RPE in AMD patients. Overall, our study demonstrates that AMBRA1-mediated autophagy may be a key process for preventing age- and stress-dependent RPE dysfunction and retinal degeneration.

## Discussion

In this study we show that a moderate reduction in autophagy, achieved through monoallelic deletion of *Ambra1* (*Ambra1^+/gt^* mice), exacerbates the retinal degeneration and visual function impairment associated with the aging process. We also describe metabolic alterations associated with mitochondrial dysfunction in *Ambra1^+/gt^* retinas that exacerbate age-dependent photoreceptor degeneration and vision loss. In the RPE, proteostasis alterations were already evident in the young *Ambra1^+/gt^* mice, resulting in RPE atrophy, increased lipid peroxidation, and the formation of lipofuscin aggregates later in life. Upregulation of *Ambra1* and of genes of the autophagy initiation machinery was also observed in the macular, but not the non-macular, region in samples from AMD patients, highlighting the protective role of autophagy in combatting disease. Lastly, *Ambra1* haploinsufficiency sensitized mice to RPE-targeted damage and subsequent photoreceptor death caused by *in vivo* sodium iodate administration, a classical model of RPE degeneration.

*Ambra1^+/gt^* mice provide a useful means of investigating how a slight reduction in autophagy, similar to which accompanies aging, impacts the metabolism and function of the retina and the RPE. As a scaffold protein, AMBRA1 is involved in a wide-range of cellular processes, including the cell cycle [48–51], tumor growth and invasion [52], cell death [53], and gene expression regulation [54]. However, most of these AMBRA1 functions occur in mitotically active tissues or cultured cells. In the retina, which is a post-mitotic tissue, AMBRA1ʹs contribution to the autophagic pathway may be of greater relevance. In line with this view, we observed no differences in MYC/c-MYC, CCND1 (cyclin D1), FOXO3 or FOXP3 expression between *Ambra1^+/gt^* mice and control littermates at any age (Figure S7), suggesting that the observed phenotype may be specifically attributed to the pro-autophagy role of *Ambra1*.

In this study, we use an outbred strain and heterozygous animals, in which some level of autophagic flux is retained. Partial loss of autophagic flux ensures a physiologically relevant context that cannot be achieved using other autophagy-deficient models, which are either perinatal lethal or, in the case of *Atg5* heterozygous animals, retain normal autophagy levels. Also, because AMBRA1 is only implicated in canonical autophagy, in contrast to ATG5 and ATG7 [55], our experimental setting eliminates the confounding effect of LC3-associated phagocytosis in photoreceptor outer segment digestion [14] and of general lysosomal deficiency [56]. Our mouse model therefore provides the unique context in which to examine how the retina-RPE ecosystem copes with the slight reduction in canonical autophagy observed during physiological aging.

The retina’s energy consumption is among the highest of all tissues, even exceeding that of the brain. The retina’s metabolic requirements are fulfilled by glucose, 80% of which is oxidized to lactate via aerobic glycolysis. These reactions produce ATP but also supply many of the building blocks, such as glyceraldehyde 3-phosphate, needed to fuel lipid biosynthesis. This is key to preserve photoreceptor and retinal function [57], as approximately 10% of POS are shed, phagocytized, and degraded daily by the RPE. Carbons from glucose also are completely oxidized to ATP in the mitochondria, as evidenced by the retina’s high level of oxygen consumption [57], and TCA cycle intermediates are also used to support anabolic reactions. These high metabolic requirements of photoreceptors are sustained by the choroidal vasculature, which delivers glucose via the RPE. The RPE is specialized in a form of energy metabolism called reductive carboxylation, which can support redox homeostasis and spares glucose for delivery to photoreceptors [58]. Our data show decreased glycolytic metabolism in the *Ambra1^+/gt^* retina, as demonstrated by reduced mRNA expression of *Hk2, Pkm*, and *Gapdh* and an increased pyruvate:lactate ratio. Reduced lactate levels increase the glucose demand of the RPE, as evidenced by the slight increase in SLC2A1/GLUT1 expression, thereby decreasing glucose delivery to the retina. Making RPE cells more glycolytic induces degeneration of neighboring photoreceptors [32,59]. In normal conditions, the retina would manage this metabolic stress by activating autophagy [60] to cope with starvation and preserve visual function, as observed in mouse models in which autophagy is selectively blocked in the RPE alone [13–15]. However, when autophagy is defective, as in *Ambra1^+/gt^* mice, the retina cannot appropriately respond to starvation by activating autophagy, thus resulting in retinal degeneration and vision defects in the long term. Mitochondrial damage and reduced glucose oxidation also result in a reduction in TCA cycle intermediates and impaired visual function [61], consistent with the marked reductions in amino acids and TCA cycle intermediates that we observed in middle-aged *Ambra1^+/gt^* retinas.

Our findings indicate that marked metabolic alterations in the retinas of middle-aged *Ambra1^+/gt^* mice impair retinal function. Purine metabolism was one of the processes in which the most significant alterations were observed. Although ATP levels were only slightly decreased, we observed a reduction in the total energy charge, in agreement with findings in cancer cells in which autophagy provides metabolic substrates to maintain energy charge and nucleotide pools [33]. Both *de novo* purine biosynthesis and the purine salvage pathway require phosphoribosyl pyrophosphate (PRPP) synthesis from ribose-5-phosphate via the pentose phosphate pathway (PPP) [62], which is highly ATP-consuming. Increases in non-phosphorylated purines and ribose-5-phosphate in old *Ambra1^+/gt^* retinas suggest that altered recycling of nucleotides in autophagy-deficient conditions may enable a compensatory increase in purine biosynthesis, which is reduced due to energy deficit. The PPP is also a source of reducing power in the form of NADPH, and we detected an increase in the NADPH:NADP ratio in old *Ambra1^+/gt^* retinas. Another possible explanation is a reduction in the consumption of NADPH for POS lipid synthesis [63,64]. Our observations suggest a pronounced bioenergetic imbalance in *Ambra1^+/gt^* retinas, in which we also observed an increase in the NADH:NAD ratio. Because NADH is mainly consumed through OXPHOS, we speculated that this finding could reflect dysfunction at the level of the mitochondrial electron transport chain. Notably, mitochondrial mass was unchanged in young *Ambra1^+/gt^* mice, although mitochondrial membrane potential was reduced. In middle-aged *Ambra1^+/gt^* retinas, we observed downregulation of the *Nqo1* gene, and of the transcription factors NRF1 and NFE2L2/Nrf2, which drive transcription of OXPHOS genes, resulting in lower mitochondrial metabolism. Overall, these results suggest alterations in the electron transport chain in the *Ambra1^+/gt^* retina. Recently, it was reported that *Ambra1* mediates PINK1 stabilization during mitophagy [65], leading to the speculation that mitochondrial alterations observed in *Ambra1^+/gt^* mice could be the consequence of impaired mitophagy.

Our findings in *Ambra1^+/gt^* retinas are also consistent with RPE damage together with alterations in proteostasis that are already evident in young animals. Indeed, deletion of other autophagy-related genes has been previously reported to induce RPE degeneration [13,43,56,66]. The RPE is a postmitotic tissue that requires efficient quality control mechanisms. Because the RPE is exposed throughout life to oxidative stress as a consequence of the visual cycle [45], autophagy plays an essential housekeeping role and facilitates the removal of intracellular debris produced by chromophore recycling [67]. Decreased autophagy has important consequences, resulting in cell enlargement and disruption of the RPE monolayer. These features can be observed in physiological aging in some geriatric mice [36], but are also seen in RPE samples from aged human patients and those with AMD [44]. We propose that *Ambra1* haploinsufficiency gives rise to RPE dysfunction that renders the RPE unable to sustain its metabolic load, ultimately leading to accelerated aging in the neural retina and vision loss. Furthermore, we show that middle-aged *Ambra1^+/gt^* mice are more sensitive to SI, which is commonly used to model RPE degeneration [37].

Our analysis of a publicly available RNA-seq dataset of retinal and RPE tissues from control and AMD patients revealed that genes of the autophagy initiation machinery, including *AMBRA1*, were markedly upregulated in the macular region of the RPE compared with the peripheral retinal region. This suggests that the AMBRA1-dependent autophagy initiation complex may be induced to counteract the stress that causes RPE dysfunction and cell loss, a key feature of macular degeneration. Disrupted metabolism was also evident. Glycolytic enzymes that were highly expressed in the retina were significantly downregulated in AMD. In RPE, the decrease in IDH2 implicates decreased mitochondrial function with AMD, which is consistent with reduced mitochondrial function reported in primary RPE cultures from human donors with AMD [42,^68^]. While our animal model does not fully replicate the cardinal features of AMD, it does inform about the retinal consequences associated with a reduction in autophagy, which has been reported in RPE tissue and primary cultures from human donors with AMD [42,43]. Taken together, these results reveal the metabolic alterations that occur in the RPE-retina in retinal degeneration and highlight the potential role of autophagy induction in preserving retinal homeostasis.

In conclusion, our findings showing that *Ambra1* haploinsufficiency exacerbates age-associated retinal degeneration indicate that maintaining autophagy is crucial to preserve metabolism and retinal function and that slight alterations in autophagy similar to those observed during normal aging promote early RPE damage. Disruption of retinal-RPE homeostasis has important consequences for visual function later in life. Therefore, interventions designed to preserve autophagy could be of therapeutic benefit to prevent blindness in the elderly as well as age-dependent retinal diseases.

## Material and methods

### Animal procedures

All animal experiments were performed following European Union guidelines and the ARVO Statement for the Use of Animals in Ophthalmic and Vision Research. Animal procedures were authorized by the CSIC ethics committee and the Comunidad de Madrid (PROEX244/17). *Ambra1* mutant mice (*Ambra1^+/gt^*) bred on a CD1 background were kindly provided by F. Cecconi [16]. In this study we used male and female *Ambra1^+/gt^* mice and *Ambra1^+/+^* littermates aged 3–4 (young), 12–15 (middle-aged), and 22–26 (old) months. The *ambra1^gt/gt^* mice were not used as these display embryonic lethality [16]. Mice were maintained at the CIB animal facility in a temperature-controlled barrier facility on a 12-h light/dark cycle, with free access to water and food. All animals were sacrificed by cervical dislocation between 9:00 a.m. and 10:00 a.m. to avoid the confounding effects of circadian rhythm changes on autophagy and retinal metabolism.

Tail bud tissue was processed to genotype *Ambra1^+/gt^* animals. Total genomic DNA was isolated using the NZY Tissue gDNA Isolation kit (Nzytech, MB13503). Polymerase chain reaction (PCR) was performed using the primers 5’-CCCAGTCACGACGTTGTAAAA-3’ (primer A) and 5’-TCCCGAAAACCAAAGAAGA-3’ (primer B), mapping downstream and upstream of the gene-trap insertion site corresponding to the lacZ reporter sequence. PCR conditions were as follows, for a total of 35 cycles: unfolding temperature, 95°C for 2 min; annealing temperature, 58°C for 54s; elongation temperature, 72°C for 45s.

For the SI model, 6.25 mg/mL of sterile sodium iodate (SI, [NaIO_3_]) solution was freshly prepared from solid SI (Sigma-Aldrich, S4007) diluted in PBS (Sigma-Aldrich, D8537). Mice received a single intraperitoneal injection of 50 mg/kg of SI solution (experimental group) or vehicle (control group) and were sacrificed 1 week after injection.

### Electroretinogram recordings

For ERG recordings mice were first allowed to adapt to darkness overnight (o/n), and then subsequent manipulations were performed in dim red light. Mice were anesthetized by i.p. injection with ketamine (95 mg/kg) and xylazine (5 mg/kg) solution while placed on a heating pad set at 37°C. Pupils were dilated with a drop of 1% tropicamide (Colircusi Tropicamida; Alcon Cusi, 57.050). A drop of 2% Methocel (Hetlingen, 8442) was placed in each eye immediately before placement of the corneal electrode to optimize electrical recording.

Flash-induced ERG responses to light stimuli produced with Ganzfeld stimulator were recorded in the right eye. A photometer was used to measure light intensity at the level of the eye (Mavo Monitor USB). In scotopic conditions, 4–64 consecutive stimuli were averaged with an interval of 10s for dim flashes and 60s for the highest intensity flashes. In photopic conditions, the interval between light flashes was set at 1 s. ERG signals were band-filtered between 0.3 and 1000 Hz and amplified with an amplifier (Grass Instruments, CP511 AC amplifier). A power laboratory data acquisition board (AD Instruments) was used to digitize electrical signals at 20 kHz. An electrode (Burian-Allen electrode; Hansen Ophthalmic Development Laboratory) was fixed on the corneal lens and a reference electrode was placed in the mouth, with a ground electrode attached to the tail to perform bipolar recordings. In dark-adapted conditions, the following responses were recorded: rod responses to light flashes of −2 log cd·s·m^−2^; and mixed responses to light flashes of 1.5 log cd·s·m^−2^. White flashes of −1.5 log cd·s·m^−2^ were used in a recording frequency range of 100–10,000 Hz to isolate the oscillatory potential (OP). In light-adapted conditions, cone-mediated responses to light flashes of 1.5 log cd·s·m^−2^ were recorded on a rod-saturating background of 30 cd·m^−2^. Wave amplitudes of scotopic rod responses (b-rod), OP, mixed responses (a-mixed and b-mixed), and photopic cone responses (b-phot and flicker) were measured off-line by an observer blind to the experimental condition of the animal. Also, the b:a wave ratio was calculated to better understand the electroretinographic responses.

### Tissue preparation for imaging

For retina and RPE/choroid flat mounts, eyes were enucleated and briefly washed in ice-cold PBS. The optic nerve, cornea, and lens were gently removed and the resulting posterior eyecup was fixed in freshly prepared ice-cold 4% (w:v) PFA (EMS, 171,010) in PBS for 30 min. Four perpendicular incisions were made and the retina and RPE/choroid were gently separated and fixed for an extra 1.5 h at room temperature (RT). Tissues were kept in 0.01% azide in PBS at 4°C.

For cryosections, mouse eyeballs were fixed o/n in 4% (w:v) PFA (171,010; EMS) at 4°C. Next, eyeballs were cryoprotected and embedded in OCT (Tissue Tek, Sakura Finetek, 4583). Sections (12 μm) were cut on a cryostat (Leica Microsystems).

## Ex-vivo *analysis of autophagic flux*

Autophagic flux was assessed as previously described [69]. Dissected retinas were placed (photoreceptors down) in Millicell support inserts (Millipore, PICM0RG50) and maintained in DMEM (Dulbecco´s Modified Eagle Medium; Gibco, 41,966–029) with 1% glutamine (2 mM; Gibco, 25.030), 1% penicillin-streptomycin (0.5 mg/mL; Gibco, 11,568,876), and 1 μM insulin (Sigma, I2643) at 37°C in a 5% CO_2_ atmosphere. Retinas were treated for 3 h with 100 μM leupeptin (Sigma, L-2884-50) and 20 mM ammonium chloride (Sigma, A9434-500) to inhibit lysosomal proteolysis. No cell death was observed during culture and there were no differences in the LC3-II:LC3-I ratio between freshly isolated retinas and *ex vivo* retinal cultures (data not shown). After culture, retinas were stored dried at −80°C.

### Cell culture

ARPE-19 cells (ATCC, CRL-2302) were grown in DMEM: F12 (1:1) supplemented with 15% FBS, 1% glutamine (2 mM; Gibco, 11,539,876), and 1% penicillin-streptomycin (0.5 mg/mL; Gibco, 11,548,876). Cells were seeded at a density of 10^5^ cells/mL in 24-well plates. For immunofluorescence, cells were grown on 12-mm glass coverslips and immunostained as previously described. For flow cytometry, cells were incubated for 30 minutes with DiOC_6_(3) (Invitrogen, D273) and Mitotracker Deep Red (MTDR; Invitrogen, M22426), DAPI (Sigma, D9542) was added to select the viable population by exclusion gating, and at least 10,000 events were recorded using a CytoFlex S system (Beckman Coulter).

### Immunofluorescence

Retinal or RPE/choroid samples were permeabilized with 2% or 0.2% (v:v) Triton X-100 (Sigma, T9284) and blocked for 1 h with BGT (3 mg/mL BSA [NZYtech, MB04602], 0.25% Triton X-100, 100 mM glycine in PBS) or block/perm solution (10% normal goat serum [Sigma, G9023], 0.1% Triton X-100 in PBS), respectively. Retinal cryosections were re-fixed with 4% (w:v) PFA for 15 min, washed in PBS, and permeabilized with Triton X-100 1% (v:v) for 1 h. Next, sections were blocked for 1 h with BGT. Samples were incubated with primary antibodies o/n at 4°C. The following primary antibodies were used: anti-LC3 (Novus, NB100-22,020; 1:200), anti-ARR3 (Millipore, AB15282; 1:200), anti-PRKCA/PKCα (Sigma, P4334; 1:200), anti- OPN1MW (Millipore, AB5405, 1:200), anti-POU4F1/BRN3A (Millipore, MAB1585, 1:100), anti-GFAP (DAKO, Z0334, 1:500), anti-GLUL (Millipore, MAB302, 1:500), anti-AIF1/IBA1 (Wako, 019–19,741, 1:500), anti-4-HNE (Abcam, ab46545, 1:100), anti-TOMM20 (Santa Cruz Biotechnology, sc-11,415, 1:200), anti-LAMP2 (ABL93, DSHB, 1:200), anti-GAPDH (Abcam, ab8245, 1:100), anti-HK2 (C64G5; Cell Signaling Technology, ab2867, 1:100), anti-SLC2A1/GLUT1 (LAB VISION, RB-9052, 1:100) and anti-AMBRA1 (Novus Biologicals, 26,190,002, 1:100). After washing with PBS, tissues were incubated for 1 h at RT in darkness with the secondary antibodies (Alexa Fluor 488, Alexa Fluor 568 and Alexa Fluor 647; Invitrogen, A11001, A11011, A21247 and A11075, 1:200) and DAPI (4’,6-diamino-2-phenylindole) (Sigma, D9542, 1 μg/mL). Additionally, RPE/choroid flat mounts were counterstained with phalloidin (A12380; Invitrogen, 1/500) together with the secondary antibodies. Finally, flat-mounted retinas were mounted with Fluoromount (Bionova, 100–01) between 2 sealed coverslips. Cryosections were mounted with DABCO (1,4-diazabicyclo[2.2.2]octane) (Sigma, D27802) and sealed with nail polish. The specificity of the LC3 antibody was verified by complete colocalization of anti-LC3 immunostaining and GFP-LC3 reporter in ARPE-19 cells (data not shown).

The ProteoStat® assay (Enzo, ENZ-51023-KP050) was performed following the manufacturer’s instructions. Cryosections were incubated with the dye at 1/500 dilution for 1 h at RT in a wet chamber before DAPI counterstaining. TUNEL assay (DeadEnd™ Fluorometric TUNEL System; Promega, G3250) was used to detect apoptotic cells in cryosections following the manufacturer’s directions. Briefly, once the primary antibody was washed, cryosections were incubated for 30 min with TUNEL buffer, after which the TUNEL reaction (1.9% TdT, 9.8% dNTPs, and 88.3% TUNEL buffer) was performed in darkness for 1 h at 37°C. Finally, saline-sodium citrate (SSC) provided in the kit (20X) was diluted to a concentration of 2X and added to cryosections to stop the TUNEL reaction.

Confocal z-stacks were obtained with Leica TCS-SP5-A0BS or Leica TCS SP8 STED 3X microscopes (Leica Microsystems). To assess retinal layer thickness, images of DAPI-stained cryosections were acquired at 40X with a Leica DMI6000B fluorescence microscope coupled to a Leica AF6000 LX multidimensional system (Leica Microsystems).

Autofluorescence was assessed using single-channel lambda-scan (xyλ) with a Leica TCS SP8 STED 3X microscope (Leica Microsystems). Briefly, cryosections were mounted using DABCO mounting medium, stimulated using the UV-laser line (λex = 405 nm) and autofluorescence was captured in 10-nm steps (λem = 420–740 nm). The RPE was manually delimited and mean fluorescence intensity was measured.

### Image analysis and data quantification

Unless stated otherwise, maximum projections of all z-stacks are displayed in representative images (z-step: 0.5 and 1 µm for RPE and retina, respectively; 1 μm for cryosections). For retinal thickness measurements, each retinal layer was manually measured using the straight-line tool in ImageJ. Quantification of mean fluorescence intensity (MFI) was performed using unprocessed images at maximal projection. Positive cells for a given marker were determined by manual counting plane by plane in a given z-stack. LC3 puncta were quantified using a manually designed Fiji-based plugin, which takes into account the 3D component. Briefly, a fluorescence intensity threshold is set to discriminate positive signal from background. Next, using a minimum voxel size threshold, the 3D objects counter tool is used to detect the number of objects in a 3D confocal z-stack. Finally, the number of puncta obtained is corrected to the thickness of the retina based on the z-stack size.

All confocal images from the same experiment were acquired using the same laser intensity and photomultiplier settings to avoid any variability or bias. Moreover, only nuclear DAPI staining was used to select the field to photograph. At least 3 sections per mice and 4 retinal regions were included in each analysis.

### Quantitative RT-PCR

Retinal RNA was extracted using TRIzol reagent (Invitrogen, 15,596–018) and converted into cDNA using the High-Capacity cDNA Reverse Transcription Kit (Applied Biosystems, 4,374,966) following the manufacturer’s instructions. Quantitative reverse transcription PCR (RT-PCR) was performed with 10 ng of cDNA in a Light Cycler® Instrument (Roche) using LightCycler® 480 probes master mix (Roche, 4,887,301,001) and Taqman probes (Life Technologies). The following TaqMan gene expression probes were used: *Sqstm1* (Mm00448091_m1), *Atg5* (Mm00504340_m1), *Wipi2* (Mm00617842_m1), *Tfeb* (Mm00448968_m1), *Ppargc1a* (Mm01208835_m1), *Nrf1* (Mm01135606_m1), *Nfe2l2 (Mm00477784_m1), Tfam* (Mm00447485_m1), *Timm23* (Hs00197056_m1), *Cox4i1* (Mm01250084_m1), *Gapdh* (Mm99999915_g1), *Hk2* (Mm00443385_m1), *Pkm* (Mm00834102_gH), *Nqo1* (Mm01253561_m1) and *Pdha1* (Mm00468675_m1). Duplicates were included in the analysis and results were normalized to expression levels of 18s RNA (Hs99999901_s).

### Western blot

Retinal protein extracts were obtained in lysis buffer consisting of 50 mM Tris-HCl, pH 6.8, 2% SDS (w:v), 10% glycerol (v:v), phosphatase inhibitors (1 mM sodium fluoride [201,154; Sigma-Aldrich], 1 mM sodium orthovanadate [S6508; Sigma-Aldrich], 5 mM sodium pyrophosphate [21,368; Sigma-Aldrich], and protease inhibitor cocktail [P8783; Sigma-Aldrich]). Isolated retinas were homogenized using a plastic pestle (Carl Roth, CXH9.1) until they were completely disaggregated. Next, samples were heated for 12 min at 95°C and stored at −20°C. Pierce BCA Protein Assay Kit (Thermo Fisher Scientific, 23,227) was used to determine protein concentration following the manufacturer’s instructions.

Total protein extract (15 μg) was mixed with 10 mM DTT and bromophenol blue and loaded into Any kD Criterion TGX Precast Gels (567–1124, Bio-Rad). PVDF membranes (170–4157, Bio-Rad) were then activated with 100% methanol and total protein extracts were transferred for 14 min at 25 V using a Trans-Blot Turbo Transfer System (Bio-Rad). Membrane-bound proteins were visualized with Ponceau S (78,376; Sigma) in 5% acetic acid (1.00063.1000; Merck). Membranes were blocked for 1 h at RT with 5% nonfat milk in PBS-T (1X PBS, 0.5% Tween 20 ([v:v] Bio-Rad, 1,706,531) and incubated o/n at 4°C with the following primary antibodies at a dilution of 1:1000: anti-LC3 (Sigma, L7543), anti-ATG12–ATG5 (Abnova, PAB12482), anti-FOXO3 (Abcam, ab70315), anti-FOXP3 (Biolegend, 320,007), anti-MYC/c-myc (Abcam, ab32072), anti-CCND1/cyclin D1 (Santa Cruz Biotechnology, sc-718), anti-TIMM23 (BD Biosciences, 611,222), anti-TOMM20 (Santa Cruz Biotechnology, sc-11,415), H2AX (Millipore, 07–164), anti-UB/ubiquitin (P4D1, Santa Cruz Biotechnology, sc-8017), anti-TUBA4A/tubulin (T6199; Sigma) anti-VCL/vinculin (Abcam, ab129002) and anti-GAPDH (Abcam, ab8245). After washing with PBS-T, membranes were incubated with the secondary antibody coupled with horseradish peroxidase (1:2000). Finally, membranes were revealed using the HRP Pierce ECL Western Blotting substrate (Thermo Scientific, 32,106) and film (AGFA, XDAOG) after exposure for varying durations. Band densitometry analysis was performed using Quantity One 1-D Analysis Software (Bio-Rad). To normalize across different gels and to encompass all 3 ages and genotypes, a sample from the ARPE-19 cell line was included in all 3 gels as an internal loading control.

### Flow cytometry of dissociated retinas

Retinas were dissected and incubated with 1% (w:v) trypsin (Gibco, 25,300,054) in DMEM for 5 min at 37°C. Each sample was centrifuged at 200 × g for 5 min and pellets were incubated with 40 nM DiOC_6_ (Invitrogen, D273) for 15 min at 37°C and 5% CO_2_ to assess mitochondrial membrane potential. Finally, dissociated retinas were resuspended in 300 μL of DMEM and analyzed in an XL flow cytometer (Beckman Coulter).

### Mass spectrometry and bioinformatics analysis

Metabolomic analyses were conducted using mass spectrometry as previously described [31]. Briefly, retinal tissue (20 mg) was obtained from 2 pools of 4 retinas from 4- and 12-month-old *Ambra1^+/gt^* mice and *Ambra1^+/+^* littermates. For each condition, tissue was weighed and solubilized with 500 μL of cold (−20°C) lysate buffer (MeOH:water:chloroform [9:1:1] with internal standards) in 1.5-mL polypropylene tubes (Precellys lysis tubes; Bertin corp., P000911-LYSK0-A). Tissue was homogenized 3 times for 20s using a Precellys 24 tissue homogenizer (Bertin Technologies). The homogenized tissue was centrifuged (10 min at 15,000 × g and 4°C) and the upper phase of the supernatant was collected. A second round of extraction was performed using 500 μL of lysate buffer, followed by homogenization and centrifugation to collect the supernatant, which was added to the first supernatant. All supernatant collected was then evaporated in microcentrifuge tubes at 40°C in a pneumatically assisted concentrator (Techne DB3). Methanol (100%, 300 μL) was added to the dried extract and the resulting solution split into 2 volumes of 150 μL each. The first part was used for the GC-MS (gas chromatography-mass spectrometry), and the second for LC-MS (liquid chromatography-mass spectrometry) analysis. The GC-MS aliquots were solubilized in methanol and transferred to a glass tube. Next, 50 μL of methoxyamine (20 mg/mL in pyridine) was added to the dried extracts, which were stored in darkness for 16 h at RT. The following day, 80 μL of N-methyl-N-(trimethylsilyl)trifluoroacetamide/MSTFA was added to perform the final derivatization at 40°C for 30 min. Samples were then transferred to glass vials and directly injected into the GC-MS system. After the second evaporation of the LC-MS aliquots, the LC-MS-dried extracts were solubilized in 50 μL of Milli-Q water, centrifuged (10 min at 15,000 × g at 4°C), transferred to glass vials, and injected into the UHPLC/MS (ultra-high-performance liquid chromatography/mass spectrometry) system. Target analysis was performed as previously described [70]. Manual verifications and QC protocols were followed to select 83 metabolites for GC targeted analysis and 17 metabolites for LC targeted analysis.

After the metabolomic analyses, a peak area proportional to concentration was obtained for each metabolite. Based on these data, metabolite ratios were calculated as the coefficient between arbitrarily chosen metabolites. Principal Component Analysis (PCA) was performed using prcomp function in R. Raw data were processed and analyzed using MultiExperiment Viewer (MEV) software. Skillings-Mack test was performed with significance set at 0.05 to compare metabolite data between *Ambra1^+/gt^* and *Ambra1*^+/+^ littermate replicates. Next, unsupervised hierarchical clustering (HCL) was performed to cluster significantly different metabolites and construct heatmap representations. Finally, the online MetaboAnalyst 4.0 tool (http://www.metaboanalyst.ca/) for metabolomic data analysis and interpretation was used to perform enrichment analysis of differentially obtained metabolites between *Ambra1^+/gt^* and *Ambra1*^+/+^ duplicates.

Processed datasets GSE142591 [40], and GSE135092 [47], were downloaded from the GEO database. FPKM (fragments per kilobase of transcript per million mapped reads) values were obtained from the already deposited data. From all detected genes in the available datasets, manually-curated autophagy or glycolysis gene lists were subtracted using R programming. Next, using pheatmap in the R package, heatmaps were generated and hierarchical clustering performed for both samples and genes. Finally, statistical analysis and plotting of selected genes was performed using GraphPad Prism software (GraphPad Software, Inc.).

### Statistical analysis

All numeric results are presented as mean values ± SEM. Statistical analyses were performed using GraphPad Prism software (GraphPad Software, Inc.). To test for differences between genotypes, two-tailed Student’s t-tests were used. To test for differences between genotypes and age class two-way analyses of variance (ANOVA) were applied and in the case of a significant interaction, differences between genotypes within each age class were assessed using Fisher’s least significant difference (LSD) post hoc tests. If assumptions of normality and homoscedasticity were not met, we applied non-parametric tests such as Mann-Whitney *U*-test for two-group comparisons and Kruskal-Wallis when more than two groups were compared. Electroretinographic responses were analyzed with general linear models using the lmer function implemented in R. The statistical model contained the electroretinographic response as response variable, genotype and sex as factors, the exact age in number of days as a covariate, the interaction between genotype and number of days, and the experimental group as random factor. Model assumptions were tested. If the normality assumption was violated, transformations were applied and in the presence of heteroscedasticity, weighted least square regressions were performed. For all tests the significance level was set at p < 0.05 (two-tailed). The number of animals used per experiment is stated in the corresponding figure legend.

## Supplementary Material

Supplemental Material

## References

[1] Léveillard T, Sahel JA. Metabolic and redox signaling in the retina. Cell Mol Life Sci. 2017;74:3649–3665. 27543457 10.1007/s00018-016-2318-7 PMC5597695

[2] Aït-Ali N, Fridlich R, Millet-Puel G, et al. Rod-derived cone viability factor promotes cone survival by stimulating aerobic glycolysis. Cell. 2015;161:817–832. 25957687 10.1016/j.cell.2015.03.023

[3] Punzo C, Kornacker K, Cepko CL. Stimulation of the insulin/mTOR pathway delays cone death in a mouse model of retinitis pigmentosa. Nat Neurosci. 2009;12:44–52. 19060896 10.1038/nn.2234 PMC3339764

[4] Sinha T, Naash MI, Al-Ubaidi MR. The symbiotic relationship between the neural retina and retinal pigment epithelium is supported by utilizing differential metabolic pathways. iScience. 2020;23:101004. 32252018 10.1016/j.isci.2020.101004 PMC7132098

[5] Kanow MA, Giarmarco MM, Jankowski CSR, et al. Biochemical adaptations of the retina and retinal pigment epithelium support a metabolic ecosystem in the vertebrate eye. Elife. 2017;6:1–25. 10.7554/eLife.28899 PMC5617631 28901286

[6] Mizushima N, Levine B. Autophagy in human diseases. In: Longo DL, editor. New England journal of medicine. Vol. 383 Boston: Massachusetts Medical Society; 2020. (16). pp. 1564–1576. doi:10.1056/NEJMra2022774. 33053285

[7] Jaeger PA, Wyss-coray T. All-you-can-eat : autophagy in neurodegeneration and neuroprotection. 2009;4(16):22. doi:10.1186/1750-1326-4-16. PMC2679749 19348680

[8] Boya P, Esteban-Martínez L, Serrano-Puebla A, et al. Autophagy in the eye: development, degeneration, and aging. Prog Retin Eye Res. 2016;55:206–245. 27566190 10.1016/j.preteyeres.2016.08.001

[9] Villarejo-Zori B, Jiménez-Loygorri JI, Zapata-Muñoz J, et al. New insights into the role of autophagy in retinal and eye diseases. Mol Aspects Med. 2021;82:101038. 34620506 10.1016/j.mam.2021.101038

[10] Rodríguez-Muela N, Koga H, García-Ledo L, et al. Balance between autophagic pathways preserves retinal homeostasis. Aging Cell. 2013;12:478–488. 23521856 10.1111/acel.12072 PMC3655122

[11] Zhou Z, Vinberg F, Schottler F, et al. Autophagy supports color vision. Autophagy. 2015;11:1821–1832. 26292183 10.1080/15548627.2015.1084456 PMC4824586

[12] Zhou Z, Doggett TA, Sene A, et al. Autophagy supports survival and phototransduction protein levels in rod photoreceptors. Cell Death Differ. 2015;22:488–498. 25571975 10.1038/cdd.2014.229 PMC4326583

[13] Zhang Y, Cross SD, Stanton JB, et al. Early AMD-like defects in the RPE and retinal degeneration in aged mice with RPE-specific deletion of Atg5 or Atg7. Mol Vis. 2017;23:228–241. 28465655 PMC5398883

[14] Kim J, Zhao H, Martinez J, et al. Noncanonical autophagy promotes the visual cycle. Cell. 2013;154:365–376. 23870125 10.1016/j.cell.2013.06.012 PMC3744125

[15] Perusek L, Sahu B, Parmar T, et al. Di-retinoid-pyridinium-ethanolamine (A2E) accumulation and the maintenance of the visual cycle are independent of Atg7-mediated autophagy in the retinal pigmented epithelium. J Biol Chem. 2015;290:29035–29044. 26468292 10.1074/jbc.M115.682310 PMC4661415

[16] Maria Fimia G, Stoykova A, Romagnoli A, et al. Ambra1 regulates autophagy and development of the nervous system. Nature. 2007;447:1121–1125. 17589504 10.1038/nature05925

[17] Di Bartolomeo S, Corazzari M, Nazio F, et al. The dynamic interaction of AMBRA1 with the dynein motor complex regulates mammalian autophagy. J Cell Biol. 2010;191:155–168. 20921139 10.1083/jcb.201002100 PMC2953445

[18] Nazio F, Strappazzon F, Antonioli M, et al. mTOR inhibits autophagy by controlling ULK1 ubiquitylation, self-association and function through AMBRA1 and TRAF6. Nat Cell Biol. 2013;15:406–416. 23524951 10.1038/ncb2708

[19] Vázquez P, Arroba AI, Cecconi F, et al. Atg5 and Ambra1 differentially modulate neurogenesis in neural stem cells. Autophagy. 2012;8:187–199. 22240590 10.4161/auto.8.2.18535

[20] Yazdankhah M, Farioli-Vecchioli S, Tonchev AB, et al. The autophagy regulators Ambra1 and Beclin 1 are required for adult neurogenesis in the brain subventricular zone. Cell Death Dis. 2014;5:e1403–8. 25188513 10.1038/cddis.2014.358 PMC4540193

[21] Dere E, Dahm L, Lu D, et al. Heterozygous Ambra1 deficiency in mice: a genetic trait with autism-like behavior restricted to the female gender. Front Behav Neurosci. 2014;8:181. 24904333 10.3389/fnbeh.2014.00181 PMC4032889

[22] Heinrich A, Nees F, Lourdusamy A, et al. From gene to brain to behavior: schizophrenia-associated variation in AMBRA1 alters impulsivity-related traits. Eur J Neurosci. 2013;38:2941–2945. 23551272 10.1111/ejn.12201

[23] Sepe S, Nardacci R, Fanelli F, et al. Expression of Ambra1 in mouse brain during physiological and Alzheimer type aging. Neurobiol Aging. 2014;35:96–108. 23910655 10.1016/j.neurobiolaging.2013.07.001

[24] Bell K, Rosignol I, Sierra-Filardi E, et al. Age related retinal Ganglion cell susceptibility in context of autophagy deficiency. Cell Death Discov. Internet]. 2020;6. Available from DOI:10.1038/s41420-020-0257-4. PMC7165178 32337073

[25] Russo R, Varano GP, Adornetto A, et al. Rapamycin and fasting sustain autophagy response activated by ischemia/reperfusion injury and promote retinal ganglion cell survival. Cell Death Dis. 2018;9. DOI:10.1038/s41419-018-1044-5. PMC6155349 30250019

[26] Mitchell SJ, Scheibye-Knudsen M, Longo DL, et al. Animal models of aging research: implications for human aging and age-related diseases. Annu Rev Anim Biosci. 2015;3:283–303. 25689319 10.1146/annurev-animal-022114-110829

[27] Fernández-Sánchez LL, De Sevilla Müller LP, Brecha NC, et al. Loss of outer retinal neurons and circuitry alterations in the DBA/2J mouse. Investig Ophthalmol Vis Sci. 2014;55:6059–6072. 25118265 10.1167/iovs.14-14421 PMC4176418

[28] Nadal-Nicolás FM, Vidal-Sanz M, Agudo-Barriuso M. The aging rat retina: from function to anatomy. Neurobiol Aging. 2018;61:146–168. 29080498 10.1016/j.neurobiolaging.2017.09.021

[29] Chang LYL, Ardiles AO, Tapia-Rojas C, et al. Evidence of synaptic and neurochemical remodeling in the retina of aging degus. Front Neurosci. 2020;14:1–16. 32256305 10.3389/fnins.2020.00161 PMC7095275

[30] Karperien A, Ahammer H, Jelinek HF. Quantitating the subtleties of microglial morphology with fractal analysis. Front Cell Neurosci. 2013;7. DOI:10.3389/fncel.2013.00003 PMC3558688 23386810

[31] Esteban‐Martínez L, Sierra‐Filardi E, McGreal RS, et al. Programmed mitophagy is essential for the glycolytic switch during cell differentiation. EMBO J. 2017;36:1688–1706. 28465321 10.15252/embj.201695916 PMC5470043

[32] Kurihara T, Westenskow PD, Gantner ML, et al. Hypoxia-induced metabolic stress in retinal pigment epithelial cells is sufficient to induce photoreceptor degeneration. Elife. 2016;5:1–22. 10.7554/eLife.14319 PMC4848091 26978795

[33] Guo JY, Teng X, Laddha SV, et al. Autophagy provides metabolic substrates to maintain energy charge and nucleotide pools in Ras-driven lung cancer cells. Genes Dev. 2016;30:1704–1717. 27516533 10.1101/gad.283416.116 PMC5002976

[34] Krohne TU, Stratmann NK, Kopitz J, et al. Effects of lipid peroxidation products on lipofuscinogenesis and autophagy in human retinal pigment epithelial cells. Exp Eye Res. 2010;90:465–471. 20059996 10.1016/j.exer.2009.12.011

[35] Fisher CR, Ferrington DA. Perspective on AMD pathobiology: a bioenergetic crisis in the RPE. Investig Ophthalmol Vis Sci. 2018;59:AMD41–AMD47. 30025108 10.1167/iovs.18-24289 PMC5989860

[36] Chen M, Rajapakse D, Fraczek M, et al. Retinal pigment epithelial cell multinucleation in the aging eye - a mechanism to repair damage and maintain homoeostasis. Aging Cell. 2016;15:436–445. 26875723 10.1111/acel.12447 PMC4854907

[37] Chowers G, Cohen M, Marks-Ohana D, et al. Course of sodium iodate–induced retinal degeneration in albino and pigmented mice. Investig Ophthalmol Vis Sci. 2017;58:2239–2249. 28418497 10.1167/iovs.16-21255

[38] MacHalińska A, Lubiński W, Kłos P, et al. Sodium iodate selectively injuries the posterior pole of the retina in a dose-dependent manner: morphological and electrophysiological study. Neurochem Res. 2010;35:1819–1827. 20725778 10.1007/s11064-010-0248-6 PMC2957578

[39] DUNN KC, AOTAKI-KEEN AE, Putkey FR, et al. ARPE-19, A human retinal pigment epithelial cell line with differentiated properties. Exp Eye Res. 1996;62:155–170. 8698076 10.1006/exer.1996.0020

[40] Tang Z, Ju Y, Dai X, et al. HO-1-mediated ferroptosis as a target for protection against retinal pigment epithelium degeneration. Redox Biol. 2021;43:101971. 33895485 10.1016/j.redox.2021.101971 PMC8099560

[41] Ferrington DA, Sinha D, Kaarniranta K. Defects in retinal pigment epithelial cell proteolysis and the pathology associated with age-related macular degeneration. Prog Retin Eye Res. 2016;51:69–89. 26344735 10.1016/j.preteyeres.2015.09.002 PMC4769684

[42] Golestaneh N, Chu Y, Xiao -Y-Y, et al. Dysfunctional autophagy in RPE, a contributing factor in age-related macular degeneration. Cell Death Dis. 2018;8:e2537–e2537. 10.1038/cddis.2016.453 PMC5386365 28055007

[43] Mitter SK, Song C, Qi X, et al. Dysregulated autophagy in the RPE is associated with increased susceptibility to oxidative stress and AMD. Autophagy. 2014;10 (11):1989–2005. doi:10.4161/auto.36184. 25484094 PMC4502658

[44] Tarau IS, Berlin A, Curcio CA, et al. The cytoskeleton of the retinal pigment epithelium: from normal aging to age-related macular degeneration. Int J Mol Sci. 2019;20. 31336621 10.3390/ijms20143578 PMC6678077

[45] Datta S, Cano M, Ebrahimi K, et al. The impact of oxidative stress and inflammation on RPE degeneration in non-neovascular AMD. Prog Retin Eye Res. 2017;60:201–218. 28336424 10.1016/j.preteyeres.2017.03.002 PMC5600827

[46] Guillonneau X, Eandi CM, Paques M, et al. On phagocytes and macular degeneration. Prog Retin Eye Res. 2017;61:98–128. 28602950 10.1016/j.preteyeres.2017.06.002

[47] Orozco LD, Chen -H-H, Cox C, et al. Integration of eQTL and a single-cell atlas in the human eye identifies causal genes for age-related macular degeneration. Cell Rep. 2020;30:1246–1259.e6. 31995762 10.1016/j.celrep.2019.12.082

[48] Chaikovsky AC, Li C, Jeng EE, et al. The AMBRA1 E3 ligase adaptor regulates the stability of cyclin D. Nature. 2021;592:794–798. 33854239 10.1038/s41586-021-03474-7 PMC8246597

[49] Maiani E, Milletti G, Nazio F, et al. AMBRA1 regulates cyclin D to guard S-phase entry and genomic integrity. Nature. 2021;592:799–803. 33854232 10.1038/s41586-021-03422-5 PMC8864551

[50] Simoneschi D, Rona G, Zhou N, et al. CRL4AMBRA1 is a master regulator of D-type cyclins. Nature. 2021;592:789–793. 33854235 10.1038/s41586-021-03445-y PMC8875297

[51] Cianfanelli V, Fuoco C, Lorente M, et al. AMBRA1 links autophagy to cell proliferation and tumorigenesis by promoting c-Myc dephosphorylation and degradation. Nat Cell Biol. 2015;17:20–30. 25438055 10.1038/ncb3072 PMC4976803

[52] Di Leo L, Bodemeyer V, Bosisio FM, et al. Loss of Ambra1 promotes melanoma growth and invasion. Nat Commun. 2021;12:2550. 33953176 10.1038/s41467-021-22772-2 PMC8100102

[53] Strappazzon F, Di Rita A, Cianfanelli V, et al. Prosurvival AMBRA1 turns into a proapoptotic BH3-like protein during mitochondrial apoptosis. Autophagy. 2016;12:963–975. 27123694 10.1080/15548627.2016.1164359 PMC4922440

[54] Schoenherr C, Byron A, Griffith B, et al. The autophagy protein Ambra1 regulates gene expression by supporting novel transcriptional complexes. J Biol Chem. 2020;295:12045–12057. 32616651 10.1074/jbc.RA120.012565 PMC7443501

[55] Martinez J, Malireddi RKS, Lu Q, et al. Molecular characterization of LC3-associated phagocytosis reveals distinct roles for Rubicon, NOX2 and autophagy proteins. Nat Cell Biol. 2015;17:893–906. 26098576 10.1038/ncb3192 PMC4612372

[56] Notomi S, Ishihara K, Efstathiou NE, et al. Genetic LAMP2 deficiency accelerates the age-associated formation of basal laminar deposits in the retina. Proc Natl Acad Sci U S A. 2019;116:23724–23734. 31699817 10.1073/pnas.1906643116 PMC6876195

[57] Ames A III. Energy requirements of CNS cells as related to their function and to their vulnerability to ischemia: a commentary based on studies on retina. Can J Physiol Pharmacol. 1992;70:S158–S164. 1295666 10.1139/y92-257

[58] Du J, Yanagida A, Knight K, et al. Reductive carboxylation is a major metabolic pathway in the retinal pigment epithelium. Proc Natl Acad Sci U S A. 2016;113:14710–14715. 27911769 10.1073/pnas.1604572113 PMC5187684

[59] Zhao C, Yasumura D, Li X, et al. mTOR-mediated dedifferentiation of the retinal pigment epithelium initiates photoreceptor degeneration in mice. J Clin Invest. 2011;121:369–383. 21135502 10.1172/JCI44303 PMC3007156

[60] Chertov AO, Holzhausen L, Kuok IT, et al. Roles of glucose in photoreceptor survival. J Biol Chem. 2011;286:34700–34711. 21840997 10.1074/jbc.M111.279752 PMC3186402

[61] Grenell A, Wang Y, Yam M, et al. Loss of MPC1 reprograms retinal metabolism to impair visual function. Proc Natl Acad Sci U S A. 2019;116:3530–3535. 30808746 10.1073/pnas.1812941116 PMC6397593

[62] Pedley AM, Benkovic SJ, Biochem T, et al. A new view into the regulation of purine metabolism-the purinosome HHS public access author manuscript. Trends Biochem Sci. 2017;42:141–154. 28029518 10.1016/j.tibs.2016.09.009 PMC5272809

[63] Rajala A, Wang Y, Brush RS, et al. Pyruvate kinase M2 regulates photoreceptor structure, function, and viability article. Cell Death Dis. 2018;9. DOI:10.1038/s41419-018-0296-4. PMC5833680 29445082

[64] Léveillard T, Philp NJ, Sennlaub F. Is retinal metabolic dysfunction at the center of the pathogenesis of age-related macular degeneration? Int J Mol Sci. 2019;20 (3):557. doi:10.3390/ijms20030557 . 30754662 PMC6387069

[65] Di Rienzo M, Romagnoli A, Ciccosanti F, et al. AMBRA1 regulates mitophagy by interacting with ATAD3A and promoting PINK1 stability. Autophagy. 2021;00:1–11. 10.1080/15548627.2021.1997052 PMC9450973 34798798

[66] Yao J, Jia L, Khan N, et al. Deletion of autophagy inducer RB1CC1 results in degeneration of the retinal pigment epithelium. Autophagy. 2015;11(6):939–953. 26075877 10.1080/15548627.2015.1041699 PMC4502815

[67] Lei Y, Liu K, Hou L, et al. Small chaperons and autophagy protected neurons from necrotic cell death. Sci Rep. 2017;7:1–13. 28720827 10.1038/s41598-017-05995-6 PMC5515951

[68] Ferrington DA, Ebeling MC, Kapphahn RJ, et al. Altered bioenergetics and enhanced resistance to oxidative stress in human retinal pigment epithelial cells from donors with age-related macular degeneration. Redox Biol. 2017;13:255–265. 28600982 10.1016/j.redox.2017.05.015 PMC5466586

[69] Esteban-Martínez L, Boya P. Autophagic flux determination in vivo and ex vivo. Methods. 2015;75:79–86. 25644445 10.1016/j.ymeth.2015.01.008

[70] Enot DP, Niso-Santano M, Durand S, et al. Metabolomic 1,2es reveal that anti-aging metabolites are depleted by palmitate but increased by oleate in vivo. Cell Cycle. 2015;14:2399–2407. 26098646 10.1080/15384101.2015.1064206 PMC4615103

