# Supplementary material for: Ambra1 haploinsufficiency in CD1 mice results in metabolic alterations and exacerbates age-associated retinal degeneration
Source: Autophagy. 2022 Jul 24;19(3):784–804. doi: 10.1080/15548627.2022.2103307 (PMC9980615; doi:10.1080/15548627.2022.2103307)
Supplement: Supplemental Material [file KAUP_A_2103307_SM4587.docx]

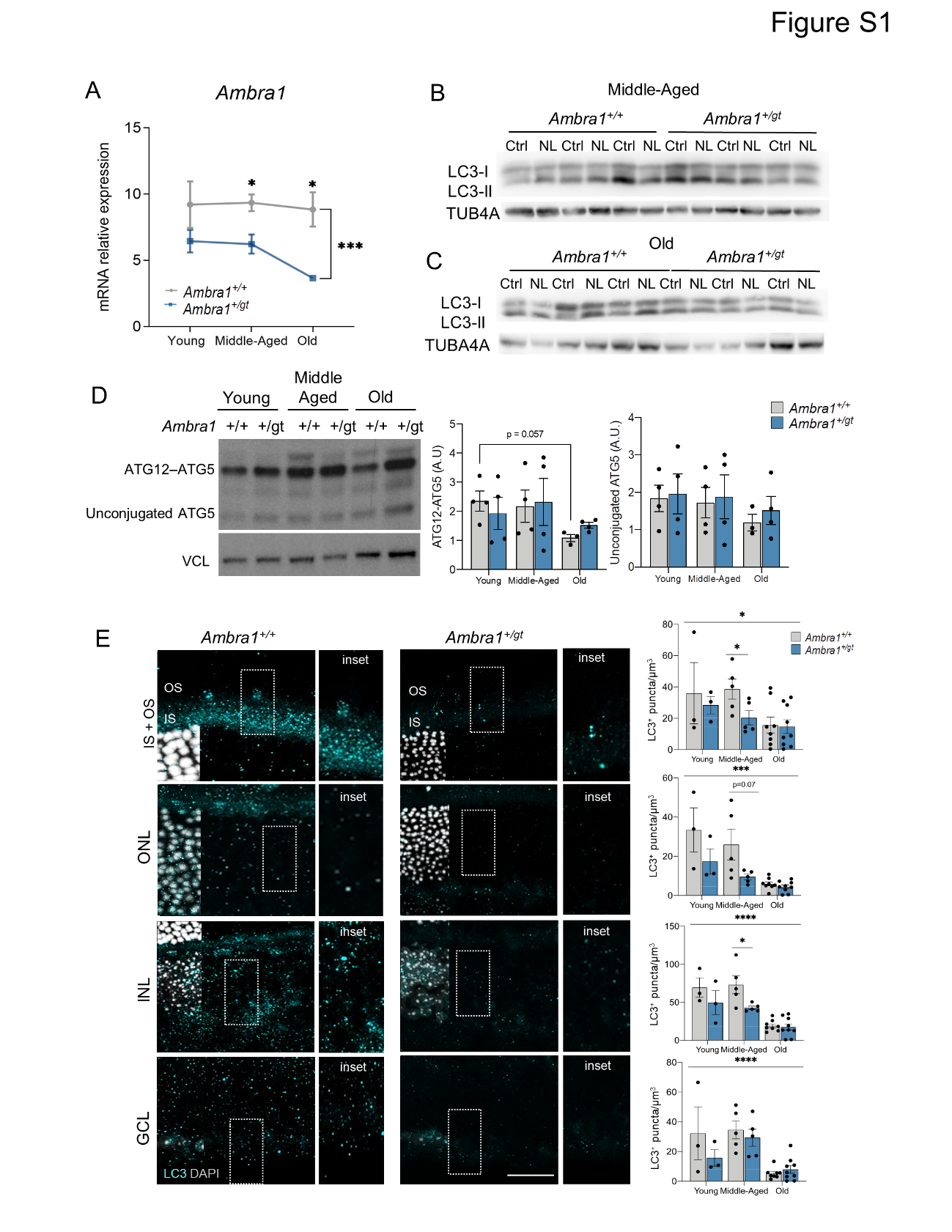


**Figure S1.** Autophagy is decreased with aging and in *Ambra1^+/gt^* mice. (**A**) Decreased *Ambra1* mRNA expression as determined by qPCR of whole retina extracts from young, middle-aged, and old *Ambra1^+/+^* and *Ambra1^+/gt^* mice (n = 3–4 per group). (**B-C**) Autophagic flux comparing *Ambra1^+/+^* and *Ambra1^+/gt^ ex vivo* retinal cultures treated with protease inhibitors (NL) with untreated retinas (Ctrl) from middle-aged (**B**) and old (**C**) mice. (**D**) Western blot showing age-related changes in the expression of the autophagy proteins ATG12–ATG5 conjugate and unconjugated ATG5 in *Ambra1^+/+^* and *Ambra1^+/gt^* mice, and corresponding quantification (right). (**E**) LC3 staining in different retinal layers. Corresponding quantification of LC3 puncta is shown on the right. OS, outer segments; IS, inner segments; ONL, outer nuclear layer; INL, inner nuclear layer; GCL, ganglion cell layer. Data are presented as the mean ± SEM. *p <0.05, ***p <0.001: two-tailed Student´s *t*-test (**E**); two-way ANOVA followed by post hoc Fischer’s LSD test for genotype (**A**) and age **(E**)**.** Scale bars: 25 µm.


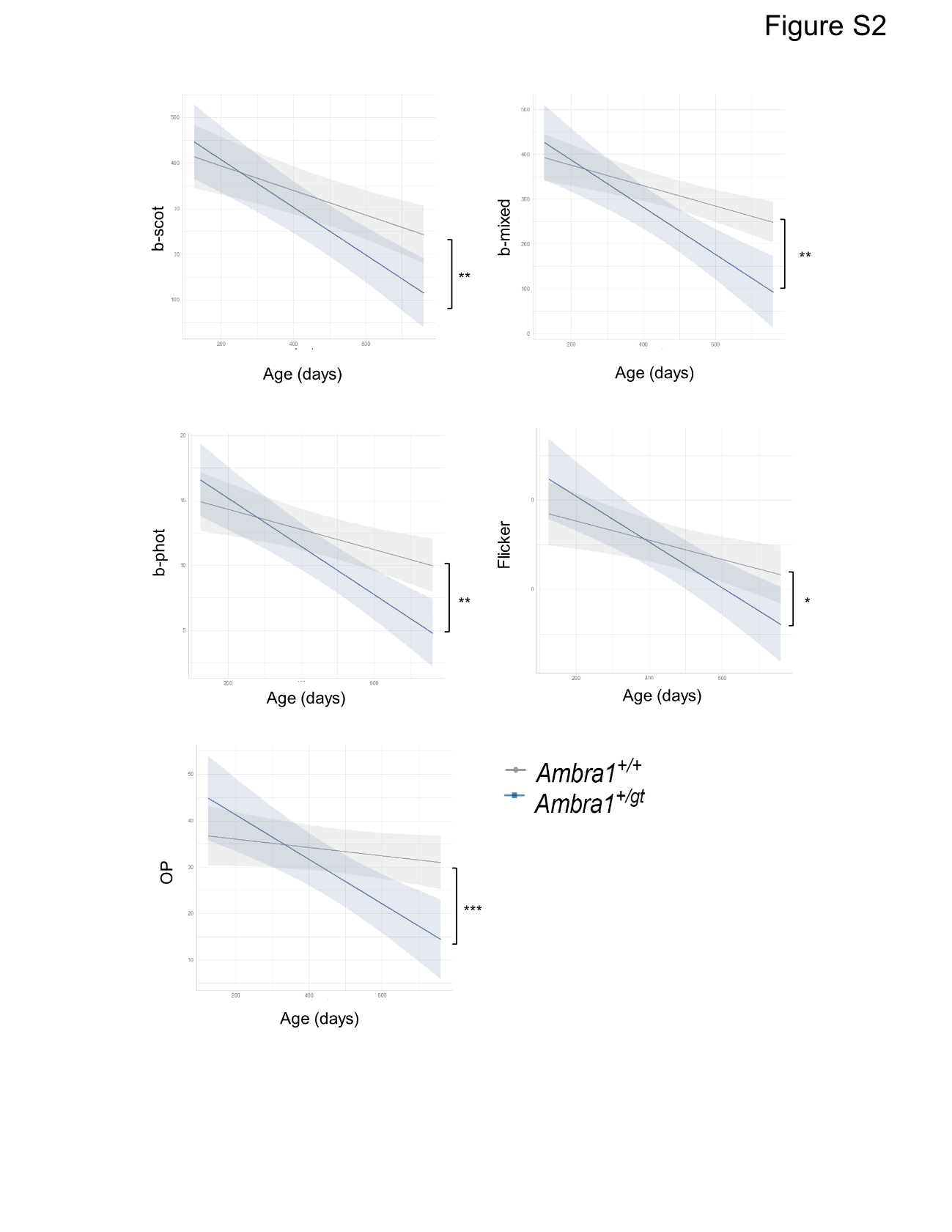


**Figure S2.** The age-associated decline in electrophysiological responses is exacerbated in *Ambra1^+/gt^* mice. (**A–E)** Electroretinographic responses measured in *Ambra1^+/+^* (n = 58) and *Ambra1^+/gt^* (n = 32) mice at different ages. The predicted regression lines and the associated 95% confidence intervals are shown. Significant slope differences are indicated: **p* <0.05, **p <0.001, ****p* <0.0001.


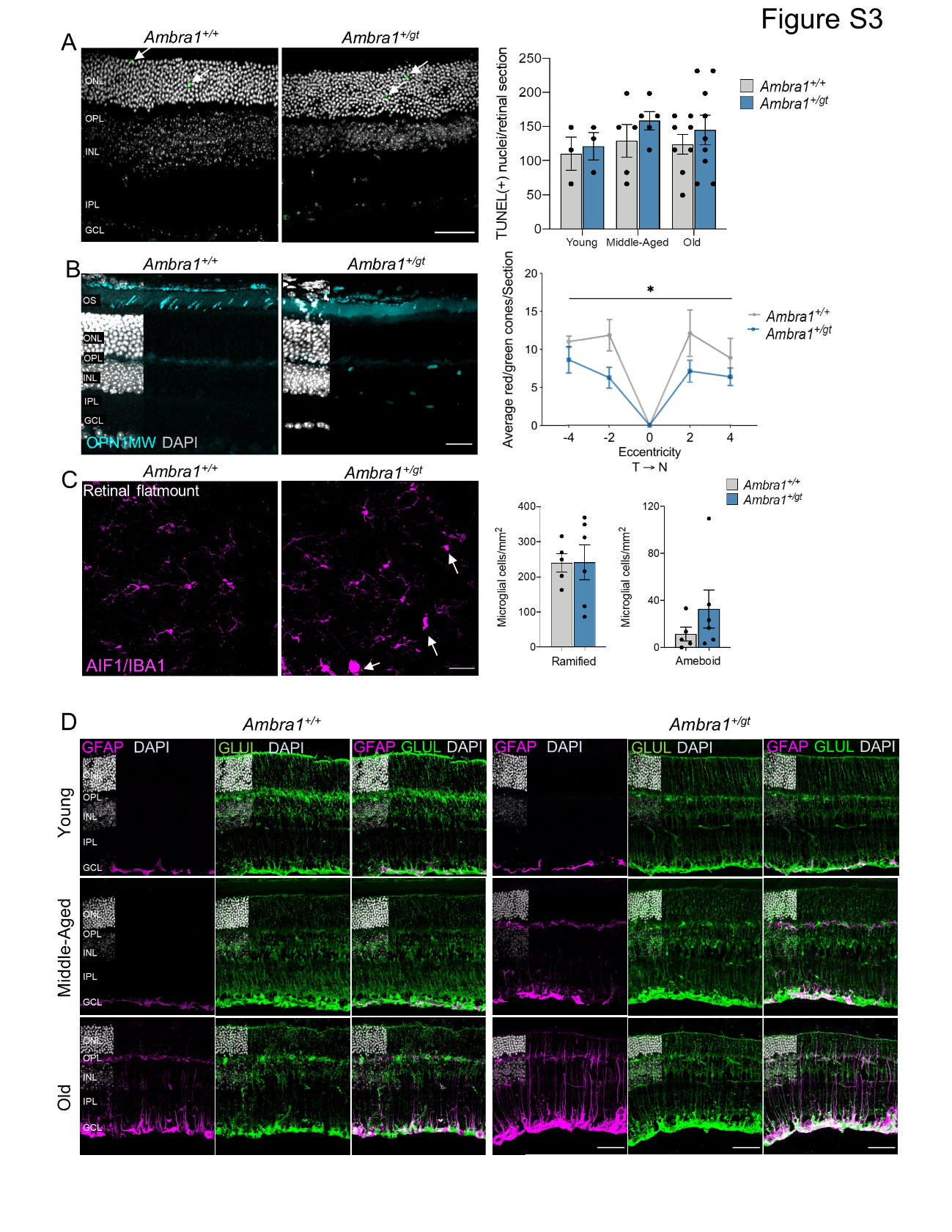


**Figure S3.** Morphological alterations and increased inflammation in old *Ambra1^+/gt^* retinas. (**A**) Detection of apoptotic cells (TUNEL, green) and corresponding quantification (right) in young, middle-aged, and old *Ambra1^+/+^* and *Ambra1^+/gt^* mice (n = 3–9). (**B**) Immunostaining of red/green cones (OPN1MW, cyan; left) and corresponding quantification (right) in temporal to nasal regions in old *Ambra1^+/+^* and *Ambra1^+/gt^* mice *(*n = 5). **(C)** Immunostaining of microglial cells (AIF1/IBA1, magenta) in retinal flat mounts (left) from old *Ambra1^+/+^* and *Ambra1^+/gt^* littermates. Two distinct microglial cell morphologies (ramified and ameboid) were observed (right) (n = 5–6 per group). Arrows indicate ameboid microglial cells. (**D**) Representative images showing immunostaining of gliosis (GFAP, magenta) and Müller cells (GLUL, green) in young, middle-aged, and old *Ambra1^+/+^* (left panels) and *Ambra1^+/gt^* mice (right panels). Nuclei are counterstained with DAPI (gray). Data are presented as the mean ± SEM. **p* <0.05: two-way ANOVA followed by Fisher’s LSD *post hoc* for genotype (**B**). Scale bars: 50 µm (**A, C** and **D**); 25 µm (**B**).


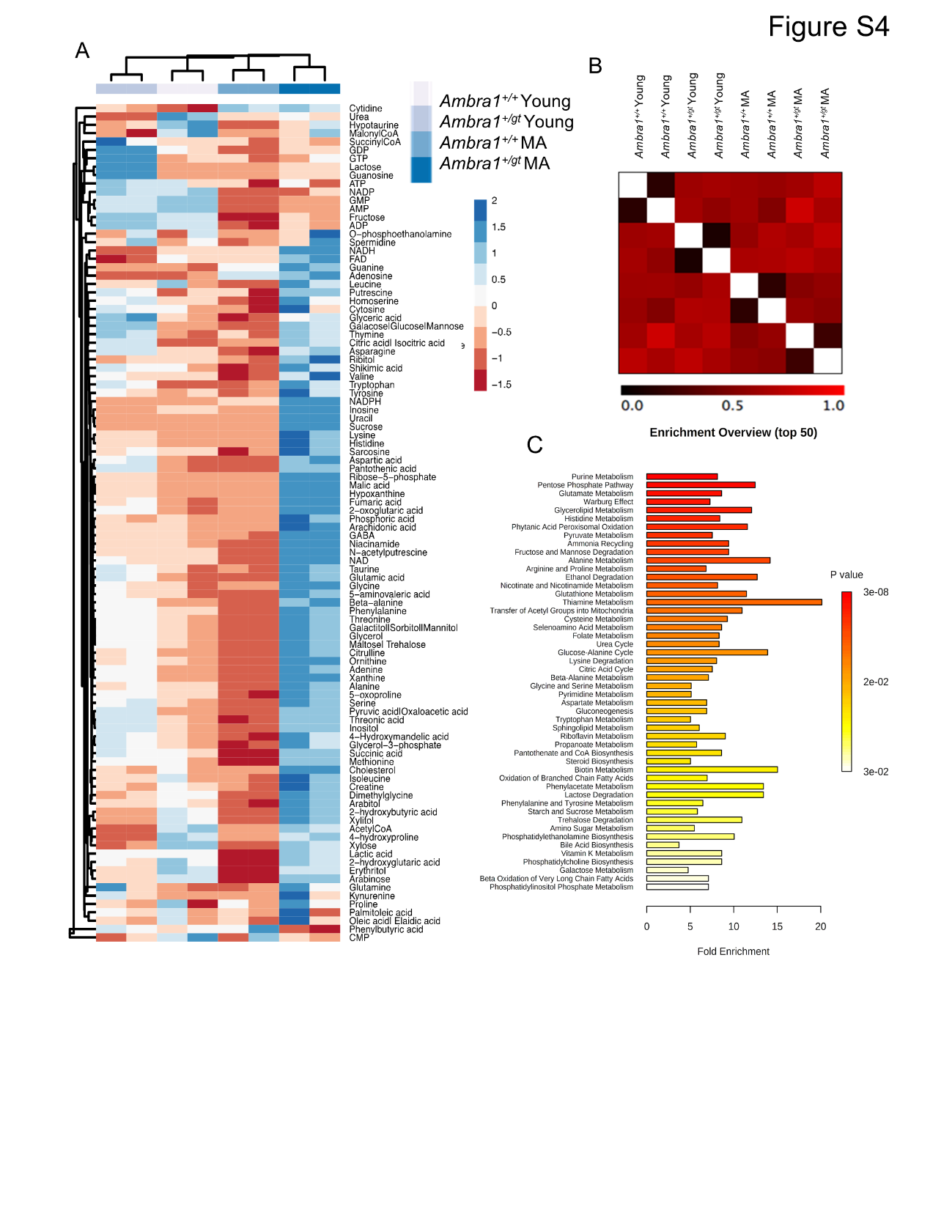


**Figure S4.** Detailed metabolomic analysis of *Ambra1^+/gt^* retinas. (**A**) Heat map and hierarchical clustering (HCL) analysis of all metabolites from young and middle-aged (MA) *Ambra1^+/+^* and *Ambra1^+/gt^* retinas (n = 2 per group). (**B**) Pairwise Pearson´s correlation of biological replicates from a metabolomic study of whole retina extracts from young and middle-aged (MA) *Ambra1^+/+^* and *Ambra1^+/gt^* mice (n *=* 2 per group). (**C**) Pathway enrichment analysis of metabolites for which significant differences were observed between *Ambra1^+/+^* and *Ambra1^+/gt^* duplicates (n = 2 per group). Statistical significance (Skillings-Mack test) was set at p <0.05.

**
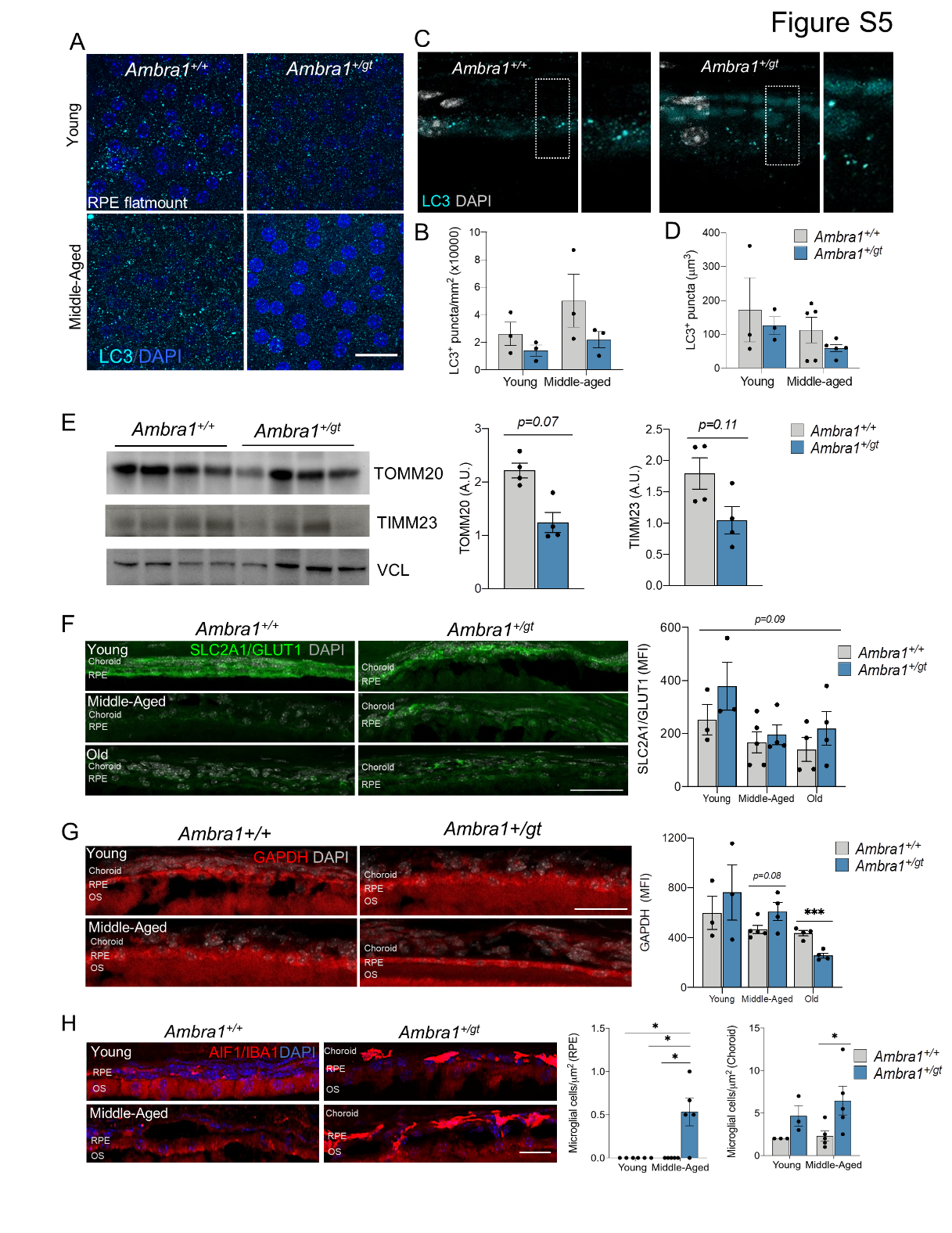
**

**Figure S5**. Middle-aged *Ambra1^+/gt^* mice show alterations in autophagy and metabolism in the RPE. (**A, B**) RPE flat mounts from *Ambra1^+/+^* and *Ambra1^+/gt^* littermates immunostained for LC3 and corresponding quantification (n = 3 per group). (**B**) Quantification of LC3^+^ puncta in **A**. (**C, D**) Immunostaining for LC3 in RPE cryosections from *Ambra1^+/+^* and *Ambra1^+/gt^* littermates and corresponding quantification of LC3^+^ puncta in young and middle-aged animals (n = 5 per group). (**E**) Levels of TOMM20 and TIMM23 proteins in the RPE were evaluated by western blot in middle-aged *Ambra1^+/+^* and *Ambra1^+/gt^* mice. (**F**) Immunostaining of SLC2A1/GLUT1 in RPE cryosections in young, middle-aged, and old *Ambra1^+/+^* and *Ambra1^+/gt^* littermates. Corresponding quantification of SLC2A1/GLUT1 mean fluorescence intensity (n = 3–5 per group). (**G**) Immunostaining of GAPDH in RPE cryosections from young and middle-aged *Ambra1^+/+^* and *Ambra1^+/gt^* littermates. Corresponding quantification of GAPDH in young, middle-aged, and old *Ambra1^+/+^* and *Ambra1^+/gt^* littermates (n = 3–5 per group). (**H**) Immunostaining of microglial cells (AIF1/IBA1, red) (left) in the RPE and choroid of young and middle-aged *Ambra1^+/+^* and *Ambra1^+/gt^* mice (n = 3–5 per group). Corresponding quantification is shown on the right. Nuclei were counterstained with DAPI (blue or gray). Data are presented as the mean ± SEM. *p <0.05; two-way ANOVA followed by Fisher’s LSD *post hoc* test for genotype (**B, D**); two-tailed Student’s *t*-test (**E**, TIMM23); or Mann Whitney *U*-test (**E**, TOMM20). Scale bars: 50 µm (**E**) and 25 µm (**A**, **F**, **G** and **H**).


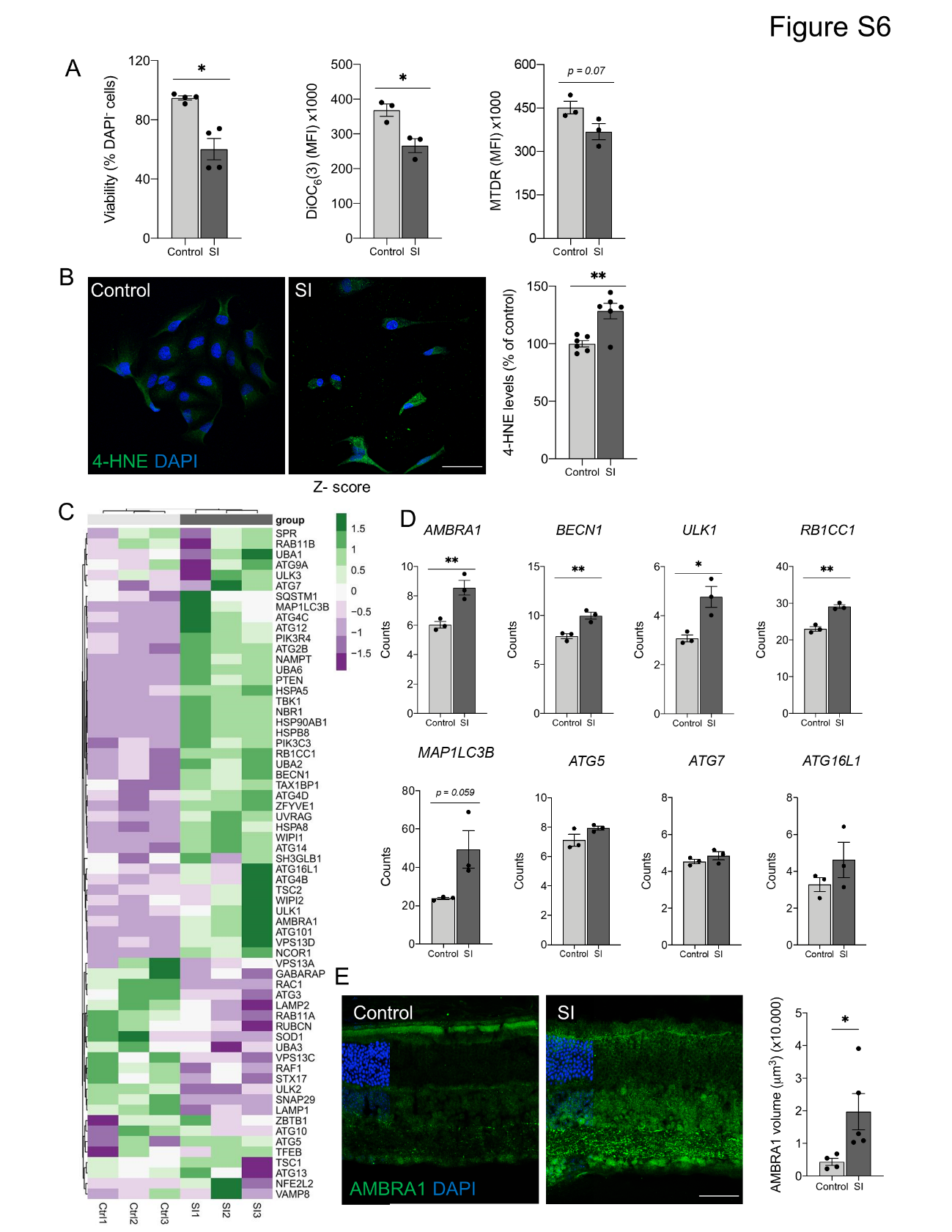


**Figure S6.** Sodium iodate (SI) induces cell death and transcription of autophagy machinery genes in ARPE19 cells. (**A**) ARPE-19 cells were treated with 20 mM SI for 24 h and then analyzed by flow cytometry to assess viability (DAPI exclusion assay), Δψm (DiOC_6_[3]), and mitochondrial mass (MitoTracker Deep Red, MTDR). n = 3–4 independent experiments with 2 biological replicates. (**B**) ARPE-19 cells were treated with 20 mM SI for 24 h and lipid peroxidation was assessed by immunofluorescence for 4-HNE (green). Nuclei were counterstained with DAPI (blue). n = 6 biological replicates from 2 independent experiments. (**C, D**) RNA-seq dataset (GSE142591) from ARPE19 cells treated with 20 mM SI for 24 h. (**C**) A manually-curated autophagy gene list was used to generate a heatmap of unsupervised hierarchical clustered samples and genes (n = 3). (**D**) Gene expression based on normalized counts of selected autophagy genes (*AMBRA1, BECN1, ULK1, RB1CC1, MAP1LC3B, ATG5, ATG7, ATG16L1*) from the same dataset (n = 3). (E) AMBRA1 immunostaining (green) and counterstained with DAPI (blue) in *Ambra1^+/+^* mice non-treated (control) or treated i.p. with SI (SI) for one week. Data are presented as the mean ± SEM. *p<0.05, **p<0.01: two-tailed Student's *t*-test (**A**, **B**, **D** and **E**). Scale bars (**A** and **E**): 50 µm.


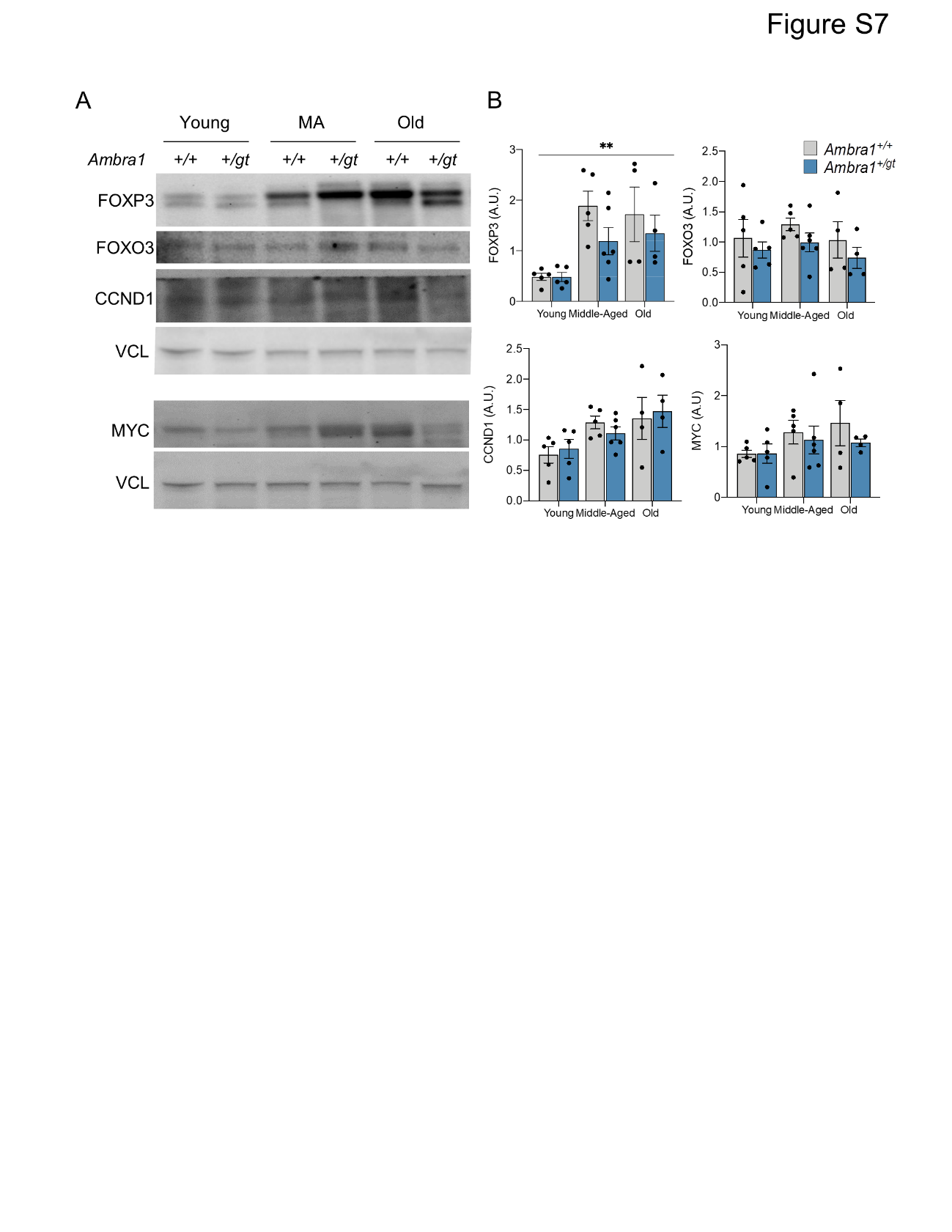


**Figure S7.** Non-autophagic functions of Ambra1 are unchanged in the RPE of *Ambra1^+/gt^* mice. (**A**) Protein levels of FOXP3, FOXO3/FOXO3A, CCND1 and MYC were evaluated by western blot in the RPE in young, middle-aged, and old *Ambra1^+/+^* and *Ambra1^+/gt^* mice. (**B**) Quantification of western blots shown in A, expressed as protein level relative to that of the loading control (VCL) (n = 4–5 per age/genotype). Statistical analysis was performed by 2-way ANOVA followed by Fisher’s LSD *post hoc* test. *p<0.05.


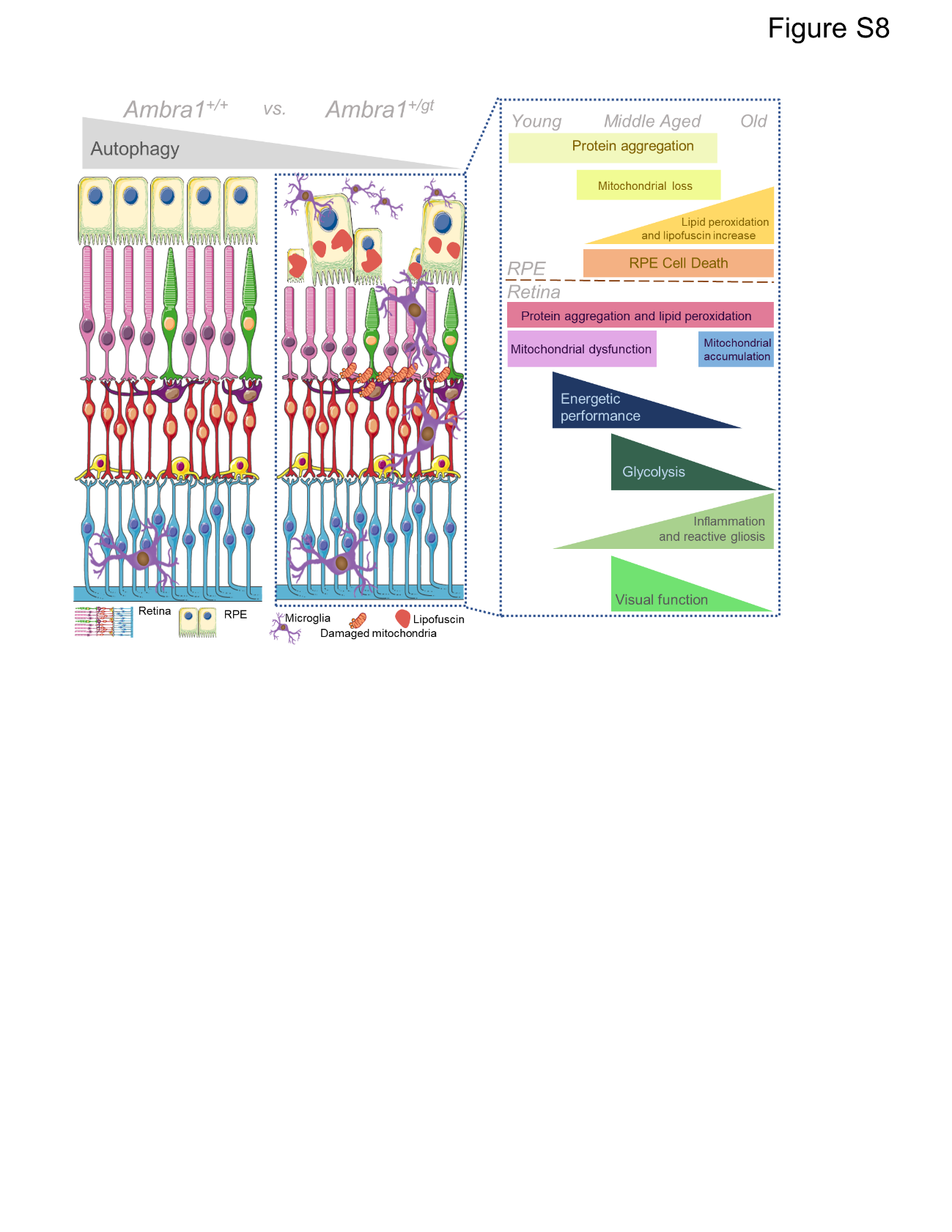


**Figure S8.** Graphical abstract. *Ambra1^+/gt^* mice display diminished autophagy activity in both the retina and RPE. This decrease in autophagy is accelerated relative to that which occurs during physiological aging. This age-associated autophagy deficiency results in premature degeneration of the RPE and retina and loss of visual function. Cellular and functional degeneration the RPE-retina is associated with protein accumulation, oxidative stress, and inflammation. Cellular damage disrupts the metabolic balance of the RPE-retina, leading to reduced mitochondrial mass in degenerating RPE cells and mitochondrial dysfunction and reduced glycolysis in the retina. Graphical abstract images templates were obtained from Servier Medical Art (<https://smart.servier.com/>).
